# A two-stages global sensitivity analysis by using the δ sensitivity index in presence of correlated inputs: application on a tumor growth inhibition model based on the dynamic energy budget theory

**DOI:** 10.1007/s10928-023-09872-w

**Published:** 2023-07-09

**Authors:** Alessandro De Carlo, Elena Maria Tosca, Nicola Melillo, Paolo Magni

**Affiliations:** 1 Electrical, Computer and Biomedical Engineering, University of Pavia, Pavia, Italy; 2 Systems Forecasting UK Ltd, Lancaster, UK

**Keywords:** Global sensitivity analysis, Uncertainty, Estimation, Correlations, TGI model, DEB Theory

## Abstract

Global sensitivity analysis (GSA) evaluates the impact of variability and/or uncertainty of the model parameters on given model outputs. GSA is useful for assessing the quality of Pharmacometric model inference. Indeed, model parameters can be affected by high (estimation) uncertainty due to the sparsity of data. Independence between model parameters is a common assumption of GSA methods. However, ignoring (known) correlations between parameters may alter model predictions and, then, GSA results. To address this issue, a novel two-stages GSA technique based on the δ index, which is well-defined also in presence of correlated parameters, is here proposed. In the first step, statistical dependencies are neglected to identify parameters exerting causal effects. Correlations are introduced in the second step to consider the real distribution of the model output and investigate also the ‘indirect’ effects due to the correlation structure. The proposed two-stages GSA strategy was applied, as case study, to a preclinical tumor-in-host-growth inhibition model based on the Dynamic Energy Budget theory. The aim is to evaluate the impact of the model parameter estimate uncertainty (including correlations) on key model-derived metrics: the drug threshold concentration for tumor eradication, the tumor volume doubling time and a new index evaluating the drug efficacy-toxicity trade-off. This approach allowed to rank parameters according to their impact on the output, discerning whether a parameter mainly exerts a causal or ‘indirect’ effect. Thus, it was possible to identify uncertainties that should be necessarily reduced to obtain robust predictions for the outputs of interest.

**Supplementary Information:**

The online version contains supplementary material available at 10.1007/s10928-023-09872-w.

## Introduction

The development process of anticancer agents is characterized by a high attrition rate [1, 2]. Although the promising results obtained during preclinical assessment, the majority of investigated compounds are withdrawn due to inadequate safety and/or efficacy in late clinical phases [3, 4]. For these reasons, taking full advantage of the information coming from preclinical studies in order to reduce the gap between the preclinical and clinical settings and to anticipate the outcomes of the clinical trials is essential [1].

Pharmacometric models can be powerful tools to summarize and quantitatively integrate information collected during the preclinical anticancer drug development, generally based on xenograft experiments, and to support their translation to the clinical setting [5–9]. The success of this model-oriented approach is witnessed by the large number of pharmacokinetic-pharmacodynamic (PK-PD) models describing the Tumor Growth Inhibition (TGI) after anticancer treatment in xenografts [5, 10–13]. PK-PD TGI models quantitatively relate plasma concentration–time curves to the tumor growth-time curves providing highly valuable metrics of tumor growth and anticancer drug activity [14, 15]. These model-derived metrics should be independent from the experimental settings and, therefore, can be successfully used to compare drug candidates, to perform preclinical-to-clinical extrapolations [16] and to anticipate the efficacious exposure to be targeted in clinics, supporting the choice of clinical efficacious doses [17, 18].

A novel mathematical model describing the tumor and host body weight dynamics during an anticancer drug treatment in xenograft mice has been recently proposed [15, 19, 20]. This model is based on the Dynamic Energy Budget (DEB) theory [21–23]. This tumor-in-host DEB-TGI model is able to integrate several aspects characterizing the in vivo TGI studies: anticancer drug activity on tumor, onset of drug-related and tumor-related cachexia and anorexia and influence of host conditions on tumor growth [24]. The incorporation of such aspects in a single model allows to investigate body weight loss due to tumor progression and treatment and, at the same time, to obtain unbiased estimates of drug anticancer efficacy. The DEB-based modeling approach was successfully applied on several in vivo preclinical studies that had been performed to assess the efficacy of the investigated compounds and that involved different host species (mice and rats), tumor cell lines, type of anticancer agents and experimental settings, including combination regimens [25]. In addition, metrics with a relevant biological meaning were derived through a mathematical analysis of the tumor-in-host DEB-TGI model in the specific formulation defined for cytotoxic agents [15]. In particular, tumor volume doubling time (TVDT), i.e., the time required by a tumor to reach a two-fold volume [26] and minimum threshold concentration necessary for tumor eradication, ${C}_{T}$, can be computed as functions of the model parameters. Interestingly, the values of TVDT and ${C}_{T}$ estimated on xenograft mice resulted predictive of the clinical settings [15] and, therefore, can be used to support the preclinical-to-clinical translation.

Uncertainty and variability are two aspects that significantly impact parameters of pharmacometric models. In the context of the DEB-TGI model, uncertainty typically refers to the estimation error introduced by the model identification procedure. This error can be particularly relevant when the experimental design and the sampling schedule are not expressly designed and optimized for model identification, thus, causing some parameters be estimated with low precision [15]. Variability is in general due to different causes. For example, in xenograft experiments, model parameters can vary among different tumor cell lines (i.e., inter-line variability), drugs (i.e., inter-drug variability) or animal species. Whatever the cause, the variation of the parameters of the model is eventually propagated to the model outputs [27]. Therefore, assessing the impact of the parameter uncertainty/variability on the final predictions of the model is a crucial step to evaluate the quality of the model-based inference. However, model parameters are generally not independent each other but they are linked by statistical dependencies (i.e., a correlation structure). Consequently, the variation of a parameter is linked to the variation of the others, according to their correlations. Thus, in this evaluation process, it is essential to take into account also the correlations between the model parameters in order to obtain a reliable analysis [28, 29].

Sensitivity Analysis (SA) and Global sensitivity analysis (GSA) are techniques aiming to investigate how the variation of the model parameters influences the model output predictions [27, 30, 31]. Both US Food and Drug Administration (FDA) and European Medicine Agency (EMA) stressed the importance of SA and GSA to support the Pharmacometrics modelling [8, 9, 32, 33]. GSA is a branch of SA which relies on the multivariate variation of all the considered input parameters [27]. GSA can be defined as *“the study of how uncertainty in the output of a model can be apportioned to different sources of uncertainty in the model input”* [27]*.* GSA allows to rank inputs (referred to as *‘factors’*) according to their impact on the output variation and to identify the model parameters whose uncertainty and variability should be reduced in order to obtain more reliable model predictions.

Several GSA methods have been proposed in the literature [34, 35]. Among them, the variance-based approach [36], which is based on the decomposition of the variance of the model output $,$ is one of the most well-established and widely used [37]. This approach is able to rank model inputs (i.e., parameters) based on their impact on model output independently on the shape of the input–output relationship, accounting also for not monotonous and nonlinear relationships. Variance-based GSA relies on the assumption of independence between model inputs [27, 36]. In such case, the variance decomposition is unique and reflects the structure of the model itself. When this hypothesis is violated (i.e., in presence of correlated inputs), the variance decomposition of model output is not unique [34]. Different extensions of the variance-based GSA method have been proposed to deal with dependent inputs [38–40], but the meaning of their results is not easy to interpret [38, 41–43].

Despite the challenges, correlations between model inputs cannot be ignored because they may dramatically alter the model predictions and, consequently, the results of SA and GSA [31, 40, 44]. In presence of statistical dependencies between inputs, the moment-independent GSA methods [31, 34] can provide a good compromise between the interpretability of GSA results and the consistency with theoretical assumptions. These techniques consider the entire distribution of the output rather than a single statistical moment (e.g., variance). In particular, the δ sensitivity index [45] describes the impact of each model parameter on the output probability density function. This metric is well-defined also in presence of statistical dependencies between the model inputs. Moreover, being a moment-independent technique, δ sensitivity index provides robust results, independently from the shape of the output distribution. Conversely, it was reported that variance-based methods can be misleading when the output distribution is multi-modal [46] or highly-skewed [47], since variance is a sensible measure of the output variation [48]*.*

In this paper, a two-stages approach for GSA exploiting the δ sensitivity index to deal with correlated inputs is presented. It was then applied to DEB-TGI model to understand the impact of the parameter estimate uncertainty on the three model-derived metrics: ${C}_{T}$, TVDT, and an additional one accounting for the trade-off between drug toxicity and efficacy. Relevant considerations with practical implications were derived from GSA results.

## Materials and methods

### Tumor-in-host DEB-TGI model

#### Description of the model structure

The tumor-in-host DEB-based model [15, 20, 24] describes the growth dynamics of the tumor and host body weight according to the DEB theory [21–23]. Briefly, it is assumed that the host body is made of two components: the energy reserves, *e(t)*, and the structural biomass, *V(t)*. The energy and structural biomass dynamics follow from energetic balances between the main physiological processes, such as the assimilation, governed by the food-supply coefficient${\rho }_{b}$, the energetic consumption and the maintenance and growth of structural biomass which costs are given by the $m$ and $g$ parameters, respectively. The tumor is conceived as an additional component able to appropriate a fraction of the host energy and to use this energy to sustain its maintenance,${m}_{u}$, and growth,${g}_{u}$, costs. The amount of energy subtracted by the tumor depends on the tumor gluttony,${\mu }_{u}$, a measure of the tumor aggressiveness. As tumor exploits host energy resource, the organism has to reduce its growth rate and, eventually, to degrade its structural biomass (tumor-related cachexia). The degradation rate increases until a certain time, ${t}_{\delta Vmax}$, when a maximum value,${\delta }_{Vmax}$, is reached. Finally, the tumor-related anorexia is modelled assuming that tumor progression inhibits host assimilation ($I{V}_{u50}$ parameter).

For cytotoxic agents, the model describes two effects: drug cytotoxicity and drug-related anorexia. Drug cytotoxicity is modelled by assuming that a fraction of tumor cells, proportional to the drug concentration through the anticancer drug potency, ${k}_{2}$, is hit by the drug and becomes not-proliferating. These cells enter into a mortality chain that is governed by the rate ${k}_{1}$. In addition, an inhibitory effect (Imax model) on host assimilation is included to account for the drug-related anorexia. The half maximal inhibitory concentration, $I{C}_{50}$, represents a measurement of drug toxicity on host body.

The complete mathematical formulation of the tumor-in-host DEB-TGI model can be found in [15, 20], while the comprehensive list of model parameter is reported in Table 1.

**Table 1:** Structural parameters of the tumor-in-host DEB-TGI model

Parameter	Units^a^	Description
Host-related parameters		
$\nu$	$L/T$	Energy conductance
${\rho }_{b}$	–	Basal food-supply coefficient
${V}_{1\infty }$	${L}^{3}$	Maximum structural biomass volume
$g$	–	Growth energy-investment ratio
$m$	$1/T$	Maintenance-growth rate ratio
$\xi$	–	Scaled reserve specific weight
${d}_{V}$	${W/L}^{3}$	Specific weight of structural biomass
${e}_{0}$	–	Initial amount of host energy reserve
Tumor-related parameters		
${\mu }_{u}$	**–**	Coefficient of gluttony
${g}_{u}$	–	Tumor growth energy-investment ratio
${m}_{u}$	$1/T$	Tumor maintenance-growth rate ratio
${d}_{Vu}$	${W/L}^{3}$	Specific weight of tumor
$I{V}_{u50}$	${L}^{3}$	Half maximal inhibitory tumor volume of food intake
Cachexia-related parameters		
$\omega$	–	Thermodynamic extraction efficiency coefficient
${\delta }_{Vmax}$	${L}^{3}/T$	Maximum degradation rate of the structural biomass
Drug-related parameters		
${k}_{1}$	$1/T$	First-order rate constant of transit for tumor mortality chain
${k}_{2}$	$CONC/T$	Drug potency
$I{C}_{50}$	$CONC$	Half maximal inhibitory concentration of food intake
Initial conditions		
${W}_{0}$	$W$	Initial host body weight
${V}_{u10}$	${L}^{3}$	Initial tumor volume

^a^L stands for length, T for time, W for weight and CONC for concentration

#### Analytical model outputs: TVDT and ${\mathrm{C}}_{\mathrm{T}}$

A mathematical analysis of the tumor-in-host DEB-TGI model allowed to define some relevant metrics quantifying tumor-in-host growth and drug anticancer activity [15]. These model-derived metrics were expressed as functions of the model parameters. Consequently, they can be easily computed once the model has been identified on experimental data without the need of performing model simulations.

First, the exponential tumor growth rate, λ, that governs the initial tumor dynamics is given by a combination of host-related and tumor-related parameters, i.e., the maintenance and growth costs of both normal and tumor cells, $m, g$ and ${{m}_{u}, g}_{u}$ respectively, along with the tumor gluttony,${\mu }_{u}:$

1 $$\lambda \, = \,\frac{{m g \mu_{u} }}{{g_{u} }}\, - \,m_{u}$$

From the exponential tumor growth rate, a model-based definition of the TVDT can be immediately derived:

2 $$TVDT\, = \,\frac{\ln 2}{\lambda }$$

Regarding the anticancer drug activity, the model postulates the existence of a concentration threshold for tumor eradication, $C{}_{T}$ (Eq. 3).

3 $$C_{T} \, = \, \frac{\lambda }{{k_{2} }}\, = \,{\raise0.7ex\hbox{${\left( {\frac{{mg\mu_{u} }}{{g_{u} }}\, - \,m_{u} } \right)}$} \!\mathord{\left/ {\vphantom {{\left( {\frac{{mg\mu_{u} }}{{g_{u} }}\, - \,m_{u} } \right)} {k_{2} }}}\right.\kern-0pt} \!\lower0.7ex\hbox{${k_{2} }$}}$$

This parameter represents the minimal (constant) concentration level beyond which tumor eradication can be asymptotically reached in xenograft mice. Consequently, it can be considered as reference concentration to be targeted in vivo for achieving a significant anticancer activity. However, keeping the drug concentration level beyond ${C}_{t}$ is a necessary but not sufficient condition for the eradication of tumor. Indeed, the animal could die before reaching the complete tumor eradication.

#### Simulation-dependent model output: ${\Delta T}_{\delta Vmax}$

**Fig. 1:**
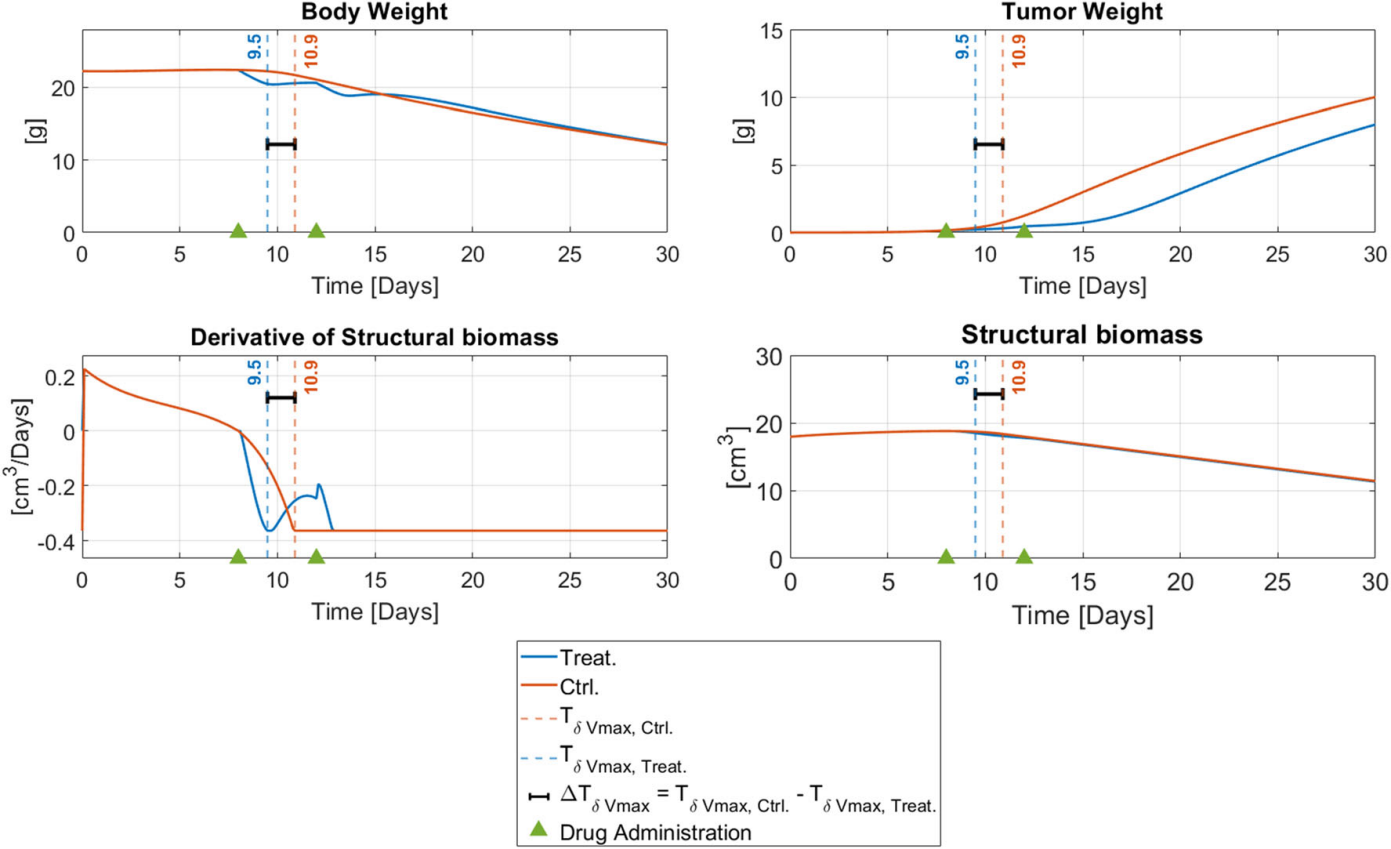
Example of ${\Delta \mathrm{T}}_{\mathrm{\delta Vmax}}$ computation. Given the schedule of administration (2 bolus of 0.5 mg/Kg at the 8th and the 12th day after tumor inoculation) and a specific parameter set of the DEB-TGI model, a simulation both in presence and absence of pharmacological treatment is performed and then ${\Delta \mathrm{T}}_{\mathrm{\delta Vmax}}$ is computed

Let us introduce an additional secondary parameter for the DEB-TGI model: the time delay, ${\Delta T}_{\delta Vmax}$, between control and treated individuals in reaching the maximum degradation rate of structural biomass, ${\delta }_{Vmax}.$ ${\Delta T}_{\delta Vmax}$, which is graphically represented in Fig. 1, is defined as

4 $$\Delta T_{\delta V\max } \, = \, T_{\delta V\max ,C} \,{-}\, T_{\delta V\max ,T} ,$$

where ${T}_{\delta Vmax, C}$ and ${T}_{\delta Vmax,T}$ represent the time at which maximum degradation rate is reached for the first time in control (untreated) and treated individuals, respectively. ${\Delta T}_{\delta Vmax}$ allows to evaluate the trade-off between toxicity and efficacy of the administered drug. The tumor exploits the host resources for its growth, inducing the degradation of the structural biomass and, consequently, the body weight loss. This tumor-related cachexia is contrasted by the drug treatment that inhibits tumor growth. However, due to its toxic effect, the drug concurrently reduces the host assimilation (drug-related anorexia), thus, worsening the degradation of host structural biomass (drug-related cachexia). Therefore, a ${\Delta T}_{\delta Vmax}>0$ underlines a toxic effect by the pharmacological treatment on the host organism as the maximum degradation rate of structural biomass is reached earlier in treated individuals. Conversely, a negative value of this index suggests that the drug inhibits tumor growth without inducing a strong reduction of the host assimilation.

Differently from ${C}_{T}$ and TVDT, ${\Delta T}_{\delta Vmax}$ has not a close analytical expression and must be calculated through model simulations. Given a drug administration schedule and model parameters values, a model simulation has to be performed both in presence and absence of drug treatment to compute ${T}_{\delta Vmax,C}$ and ${T}_{\delta Vmax,T}$. In this work, NONMEM software tool was exploited for performing simulations. The set-up is summarized in the Table S1.1 of the Supplementary Material S1 which also contains the NONMEM code.

### Two-stages GSA with δ sensitivity index

#### δ sensitivity index

Let us consider a generic model

5 $$Y\, = \,g\left( {\varvec{X}} \right)$$

where $Y$ is the scalar output of the model, $g$ represents the inputs-output relationship and ${\varvec{X}}=\left\{{X}_{1},\dots ,{X}_{N}\right\}$ is the ${R}^{N}$ vector of the input parameters. Within the GSA framework, ${\varvec{X}}$ is considered as a random variable [27] which is characterized by a joint probability density function (pdf)$, {f}_{{\varvec{X}}}\left({\varvec{X}}\right).$ Therefore, $Y$ is a random variable with a pdf, ${f}_{Y}(Y)$, which can be calculated through Eq. (5) using input samples extracted from ${f}_{{\varvec{X}}}\left({\varvec{X}}\right).$

The definition of the δ index relies on the following considerations [45]. Suppose that one input ${X}_{i}$ can be fixed to a certain value ${x}_{i}^{*}$, then, the conditional pdf of $Y$ given ${X}_{i}={x}_{i}^{*}$, ${f}_{Y|{X}_{i}}\left(Y|{X}_{i}={x}_{i}^{*}\right),$ can be defined. The shift between ${f}_{Y}(Y)$ and ${f}_{Y|{X}_{i}}\left(Y|{X}_{i}={x}_{i}^{*}\right)$ can be measured as

6 $$s\left( {X_{i} } \right)\, = \,\smallint |f_{Y} \left( Y \right)\, - \,f_{Y} (Y|X_{i} )|dY$$

**Fig. 2:**
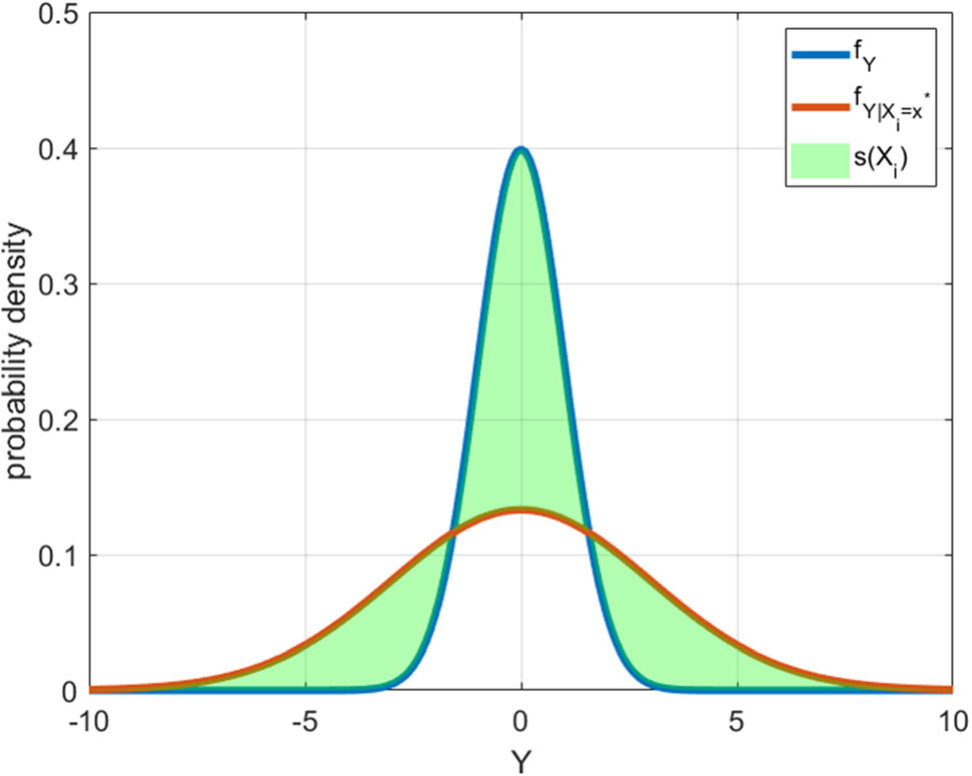
Comparison between conditional and unconditional pdf of Y. The difference s(${\mathrm{X}}_{\mathrm{i}}$) between these two distributions is represented by the green shaded area

As illustrated in Fig. 2, $s\left({X}_{i}\right)$ represents the difference of the area underlying ${f}_{Y}(Y)$ and ${f}_{Y}(Y|{X}_{i})$, which corresponds to the impact of fixing ${X}_{i}$ to ${x}_{i}^{*}$ on ${f}_{Y}(Y)$. ${X}_{i}$ is a random variable typically assuming more values than just ${x}_{i}^{*}$. The $\delta$ sensitivity index for ${X}_{i}$ can be computed through the expected value of $s\left({X}_{i}\right)$ over the entire domain of ${X}_{i}$ as in Eq. (7), where ${f}_{{X}_{i}}({X}_{i})$ is the marginal pdf of ${X}_{i}$.

7 $$\delta_{i} \, = \,\frac{1}{2}E_{{X_{i} }} \left[ {s\left( {X_{i} } \right)} \right]\, = \,\smallint f_{Xi} \left( {X_{i} } \right)\left[ {\smallint \left| {f_{Y} \left( Y \right)\, - \,f_{{Y|X_{i} }} \left( {Y|X_{i} } \right)} \right|dY} \right]dX_{i}$$

It has been demonstrated in [45] that $0\le {\delta }_{i}\le 1$, in particular ${\delta }_{i}=0$ if and only if $Y$ is independent from ${X}_{i}$ and ${X}_{i}$ is uncorrelated from the other ${X}_{j},$ with $i\ne j$ A full description of the properties of this sensitivity index can be found in [45].

#### Two-stages GSA approach

Although the δ sensitivity index is well-defined also in presence of correlations among the model inputs [45], the interpretation of the GSA results remains challenging.

As an example, let us consider a simple linear model, $Y=g\left({X}_{1},{X}_{2}{,X}_{3}, {X}_{4}\right)=100*{X}_{1}+100*{X}_{2}+{10*X}_{3}$, with ${X}_{i}\sim N(\mathrm{0,1})$ and let ${X}_{4}$ be correlated to ${X}_{1}$ with ${\rho }_{\mathrm{1,4}}=0.9.$ From the equation defining Y, it is evident that (1) ${X}_{1}$ and ${X}_{2}$ are the most impactful terms on $Y$ due to their high coefficients; (2) the spurious term ${X}_{4}$ does not exert a causal effect because it does not directly appear in the output equation and (3) ${X}_{4}$ can exert an ‘indirect’ effect on $Y$ due to its correlation with ${X}_{1}$. Now, let us perform a GSA and compute the δ indices for the four model inputs first neglecting and then, accounting for the correlation between ${X}_{1}$ and ${X}_{4}$. As shown in Figure S2. 1 and S2.2 in the Supplementary Material S2, the δ indices for ${X}_{1}, {X}_{2}$ and ${X}_{3}$ assume the same positive value in both the cases with ${\delta }_{{X}_{1}}$= ${\delta }_{{X}_{2}}$>${\delta }_{{X}_{3}}.$ Differently, the value of the δ index for ${X}_{4}$ varies in the two situations. When ${\rho }_{\mathrm{1,4}}$ is neglected, ${\delta }_{{X}_{4}}=$ 0 and, therefore, it is impossible to quantify the ‘indirect’ impact of ${X}_{4}$ on $Y$. Instead, when ${\rho }_{\mathrm{1,4}}$ is considered, ${\delta }_{{X}_{4}}$ assumes a positive value higher than ${\delta }_{{X}_{3}}$ and comparable to ${\delta }_{{X}_{1}}$ and ${\delta }_{{X}_{2}}$, so that, in this case, it is difficult to distinguish which model inputs have a causal impact on the output.

From this simple example, it is clear that, although considering the correlations between the model parameters is essential for a correct evaluation of their impact on the model output [28, 40], it could make hard the identification of the model parameters exerting a causal effect. Therefore, it is necessary to perform also the GSA without considering the statistical dependencies between model inputs as it allows to easily detect parameters with a direct impact [49].

Based on these considerations, a two-stages approach to perform a GSA with δ index in presence of correlated inputs is here proposed. The analysis is composed by two subsequent steps. In Step 1, a first set of δ indices, $\delta_{1,i} ,$ is computed under the hypothesis of independence between model parameters so that the parameters exerting a causal effect on ${f}_{Y}(Y)$ can be easily identified. In Step 2, a new set of indices, ${\delta }_{2,i}$, is computed considering the statistical dependencies between model inputs. In this way, the ‘correct’ ${f}_{Y}(Y)$ is considered and the ‘indirect’ contributions of each ${X}_{i}$ on the output distribution due to the correlations with the other parameters is accounted for. At the end, each parameter ${X}_{i}$ of the model is characterized by two indices, ${\delta }_{1,i}$ and ${\delta }_{2,i}$. From their values it is possible to establish that:

- ${X}_{i}$ has a causal effect if ${\delta }_{1,i}>0$, whereas it is a spurious input if ${\delta }_{1,i}=0.$
- ${X}_{i}$ has only an indirect effect if ${\delta }_{1,i}=0$ and ${\delta }_{2,i}>0$. In this case ${\delta }_{2,i}$ represents only the impact of ${X}_{i}$ due to its correlations with other model parameters as in the Step 1 emerged that it can be considered a ‘spurious’ term with respect to the model output.
- ${X}_{i}$ has both a causal and indirect effect if ${\delta }_{1,i}>0$ and ${\delta }_{2,i}>0$. In this case, ${\delta }_{2,i}$ represents the impact of ${X}_{i}$ due to its variation and its correlations with other model parameters.

### GSA of the DEB-TGI model

#### Distribution of model parameters

Within the GSA framework, the model parameters are considered as random variables characterized by a joint pdf accounting for their variability/uncertainty.

The tumor-in-host DEB-TGI model is generally identified in subsequent steps [15]. First the tumor-related parameters are estimated on healthy (tumor-free) mice growth data; then, the tumor-related, cachexia-related and drug-related parameters are simultaneously identified on tumor and host body weight data from xenograft animals keeping fixed the parameters related to the host and to the PK model.

The aim of this work is to evaluate the impact of the parameter uncertainty coming from the model identification on experimental data from xenograft studies. Thus, it was necessary to define a joint pdf only for the model parameters that are typically estimated on xenograft data (Table 2). Differently, the parameters related to the host and to the PK model, as well as ${d}_{Vu}, {e}_{0}$ and ω, that were typically fixed to the values reported in the Table S3. 1 in the Supplementary Material S3, were not considered. In particular, a two-compartment model with linear elimination was adopted to describe drug PK.

**Table 2:** Typical values of DEB-TGI model parameters identified on experimental data together with their CVs

Parameter	Value	Unit	CV1 [%]	CV2 [%]
${\mu }_{u}$	13.321	–	5	50
${g}_{u}$	10.607	–	5	50
${m}_{u}$	1.459e-2	$1/day$	30	80
${\delta }_{Vmax}$	4.146e-2	–	20	60
${V}_{u10}$	1.160e-3	$g$	20	60
${W}_{0}$	22.263	$g$	2	5
${R}_{b}$	7.310e-2	–	20	80
$I{V}_{u50}$	5.300	$c{m}^{3}$	10	60
${k}_{1}$	0.381	$1/day$	30	80
${k}_{2}$	0.129	$ml/ng day$	20	40
$I{C}_{50}$	2.530e-2	$ng/ml$	10	60

Two scenarios were considered depending on estimation accuracy: CV1 high accuracy, CV2 low accuracy. CVs of parameters are expressed as percentages

Due to their biological meaning (see Table 1), all the parameters can assume only positive values. Thus, a multivariate lognormal distribution, $LogN({\varvec{\theta}},{\varvec{\Omega}})$ with mean **θ** and variance–covariance matrix **Ω**, was used to characterize the joint pdf of the considered model parameters. The typical values, ${\varvec{\theta}},$ were taken from the parameter estimates obtained by fitting the model on preclinical data [15] and reported in Table 2. Because in the model estimation, the food-supply coefficient ${\rho }_{b},$ is parametrized as

8 $$\rho_{b} \, = \,\frac{1}{{1\, + \,R_{b} }}$$

${R}_{b}$ was considered in the GSA.

In the first stage of the GSA, parameter variances were selected based on the coefficients of variation (CVs) of the estimates uncertainty and no correlation was considered [15, 19]. In particular, two scenarios were analysed to account for situations of accurate (CV1, lower values) and of less accurate (CV2, higher values) estimates.

In the second stage, correlations between model parameters were introduced based on typical correlation matrices obtained during model identification (internal data, not shown): parameter couples were classified in significantly correlated (with corr =  ± 0.95, 0.75 or 0.6), uncorrelated (corr = 0) or with an unknown correlation, as illustrated in Figure S3. 1 in the Supplementary Material S3. In particular, the last class included parameters couples for which correlations spanned over a wide range of values depending on the considerd experimental data.

**Fig. 3:**
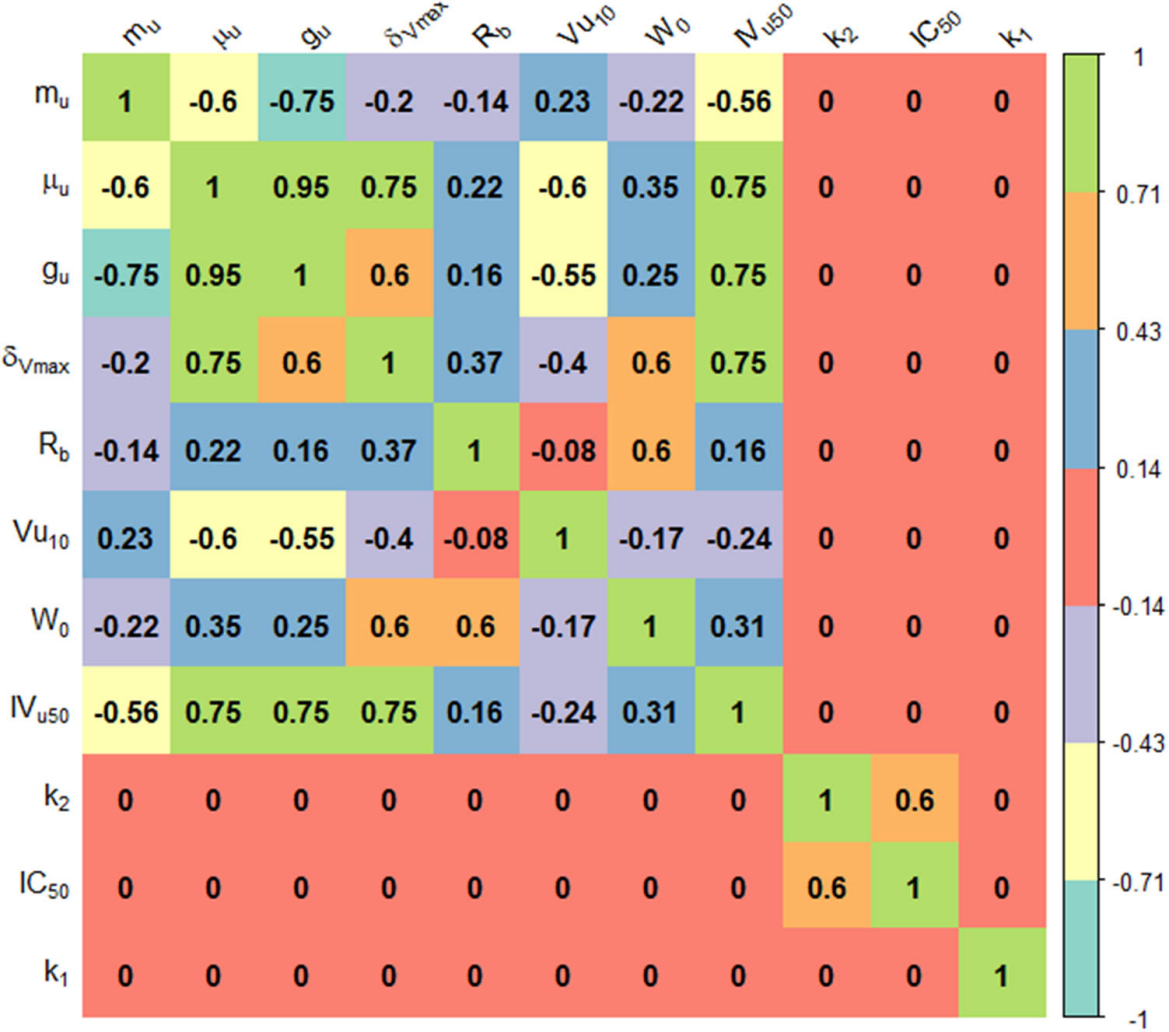
Correlation matrix for CV1 scenario obtained after the estimation of unknown correlations with the R package mvLognCorrEst

The R package *mvLognCorrEst* [50] was used to estimate unknown correlations. The correlation structures obtained for the CV1 and CV2 scenarios are reported in Fig. 3 and Figure S3. 2 of the Supplementary Material S3, respectively. In particular, the Supplementary Material S3 provides a detailed description of how the correlation structure was built in the CV2 case.

### Preliminary uncertainty analysis

A preliminary uncertainty analysis [27] was performed for each model output, $Y$, using a Monte Carlo simulation approach [34]. The uncertainty analysis was executed both in presence and absence of correlations between the model parameters. This was done to assess the changes in ${f}_{Y}(Y)$ when statistical dependencies are neglected.

100,000 samples were extracted from the joint lognormal distribution of ${\varvec{X}}$ for both CV1 and CV2 scenarios. Then, for each extracted parameters set, the output of interest was computed. Eqs. (2) and (3) were used for TVDT and ${C}_{T}$ respectively, while ${\Delta T}_{\delta Vmax}$, was calculated as explained in the section “*Simulation-dependent model output: *${\Delta \mathrm{T}}_{\mathrm{\delta Vmax}}$*”.*

#### Implementation of the two-stages GSA

After the preliminary uncertainty analysis, the Two-stages GSA approach, here proposed, was implemented. A numerical “given data” approach to estimate the δ sensitivity index has been proposed by Plischke et al*.* [51]. This strategy leverages Eq. (7) to compute the δ index and the kernel density estimation to characterize both the pdf of the output, ${f}_{Y}(Y)$, and the conditional pdf of the output given an input ${X}_{i},$ ${f}_{Y}\left(Y|{X}_{i}\right)$. In this work, the MATLAB implementation of the numerical approach presented in [51] and shared by the authors was used.

For each model output, variability scenario and step of the GSA, 100,000 samples of model parameters were extracted from the joint lognormal distribution of inputs (with or without correlations depending on the step of the GSA) using a pseudorandom strategy (*rand* MATLAB function [52]). For each parameter set the corresponding value of the output was computed as previously detailed. All the input–output sets were used to calculate sensitivity indices. Uncertainty of the sensitivity indices was calculated with 1000 bootstrap samples [53].

Moreover, in each step of the GSA, a spurious input (i.e., not belonging to the DEB-TGI parameters and uncorrelated from them) extracted from a $N\left(\mathrm{0,1}\right)$ and named ‘Noise’, was included in the analysis. ${\delta }_{Noise}$ was introduced as negative control to provide a quantitative evaluation of the noise/bias introduced in the sensitivity indices estimation by their numerical computation [51]. The 97.5th percentile of ${\delta }_{Noise}$ was used as threshold value for evaluating whether ${\delta }_{i}$ can be considered different from zero: in case the median ${\delta }_{i}$ for ${X}_{i}$ was lower than the 97.5th percentile of ${\delta }_{Noise}$, ${\delta }_{i}$ was considered equal to zero.

## Results

### Results for ${C}_{T} \text{ and }TVDT$

**Fig. 4:**
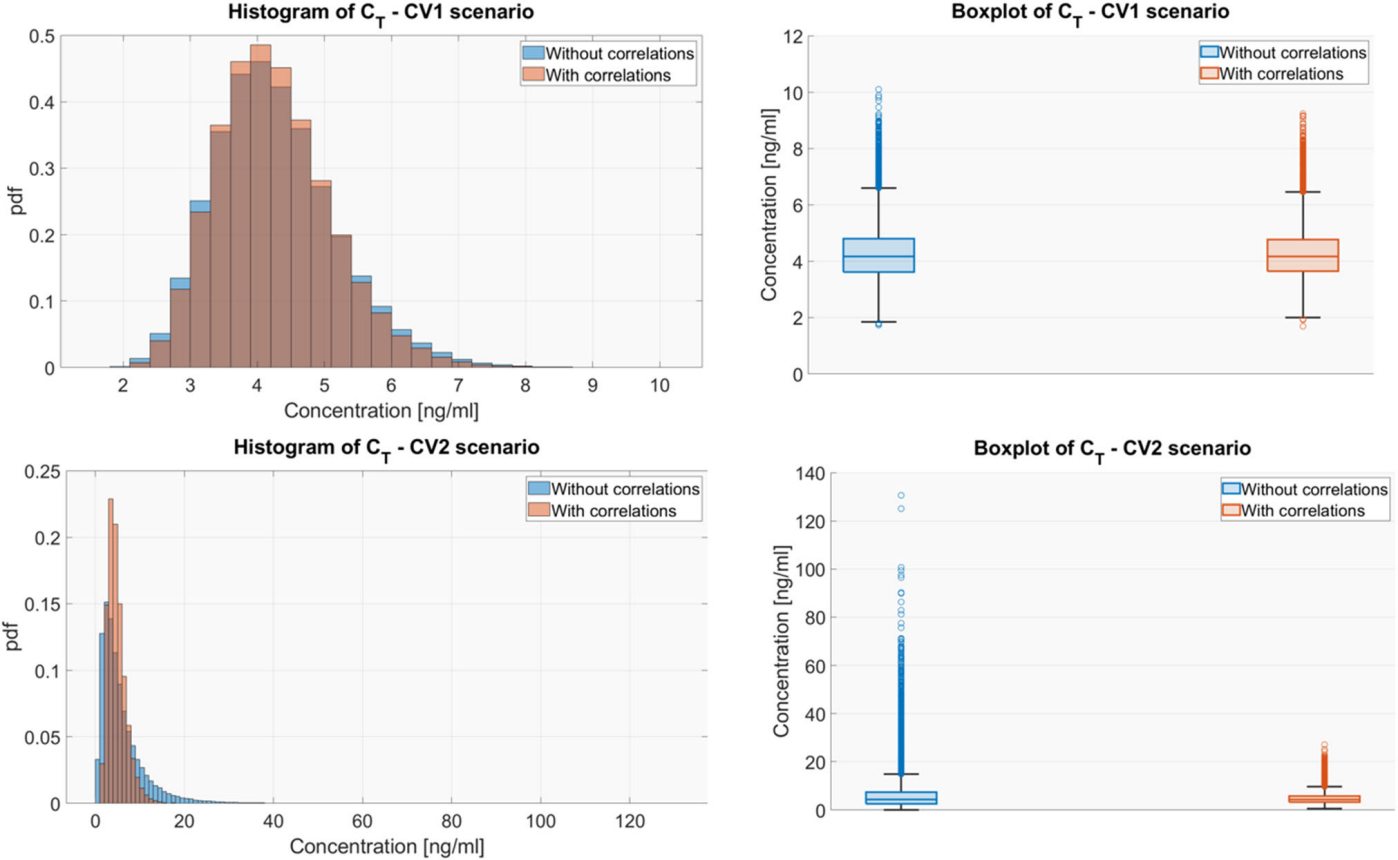
Histograms and box plots of ${\mathrm{C}}_{\mathrm{T}}$ considering and ignoring correlations for each uncertainty scenario

In Fig. 4 and Figure S4. 1 of the Supplementary Material S4 the uncertainty distributions of ${C}_{T}$ and TVDT are reported. From these figures, it is possible to appreciate the dramatic impact that ignoring the parameter correlations can exert on the output distribution. In particular, neglecting the correlations led to an overestimation of the output uncertainty. This overestimation is quite moderate when the parameter CVs are low: for CV1 the 95% CI of ${C}_{T}$ is [2.82, 6.15] and [2.75, 6.33] *ng/ml* and for TVDT is [1.27, 1.35] and [1.13, 1.51] *day* in case of presence and absence of correlations, respectively. Conversely, the overestimation of the uncertainty becomes relevant in the scenario with high CVs: for CV2 the 95% CI of ${C}_{T}$ is [1.94, 10.06] and [0.90, 20.37] ng/ml and of TVDT is [0.94, 1.82] and [0.34, 5.28] day in case of presence and absence of correlations, respectively. Interestingly, when the correlations between model parameters are considered, the ranges of ${C}_{T}$ and TVDT approximately remain within two folds of the means, even in the CV2 scenario where the model parameters are characterized by a high uncertainty. Therefore, the estimation uncertainty has a limited effect on the variation of the two model-derived metrics.

**Fig. 5:**
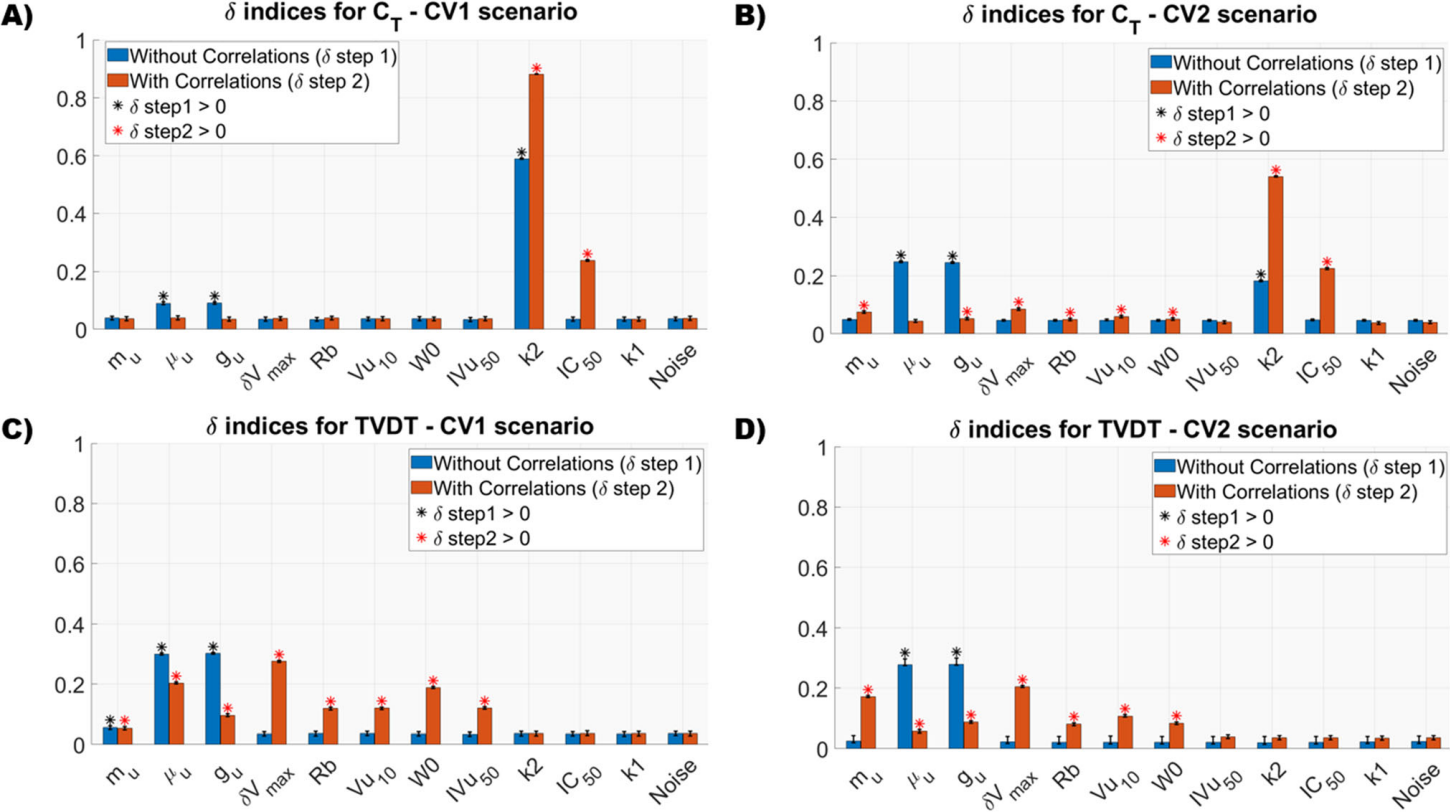
Results of the Two-Stages GSA on ${\mathrm{C}}_{\mathrm{T}}$ and TVDT in both CV1 and CV2 scenario. Black and red asterisks mark parameters with a δ significatively greather than 0 in Step 1 and Step 2, respectively

The results of the proposed two-stages GSA are summarized in Fig. 5.

Panels A and B show $\delta$ indices obtained for ${C}_{T}$ in the CV1 and CV2 scenarios, respectively. In the CV1 scenario, in absence of correlations (Step 1), the drug potency, ${k}_{2}$, resulted the parameter with the highest causal impact on the ${C}_{T}$ distribution, followed by moderate effect of the tumor-related parameters ${\mu }_{u}$ and ${g}_{u}$. As expected, ${\delta }_{Vmax}$, ${R}_{b}$, ${V}_{u10}$, ${W}_{0}$, ${IV}_{u50}$, ${IC}_{50}$ and ${k}_{1}$, that do not appear in the ${C}_{T}$ definition (Eq. (3)), did not have a direct impact (${\delta }_{1}$=0) on the model output. However, when the correlation is considered (Step 2) ${IC}_{50}$ gained an ‘indirect’ impact due to its correlation with ${k}_{2}$ (Fig. 3). Interestingly, although ${m}_{u}$ appears in the definition of ${C}_{T}$ and is affected by a high uncertainty (Table 2), its $\delta$ was equal to 0 in both steps. In the CV2 scenario (panel B of Fig. 5), the tumor growth related parameters ${\mu }_{u}$ and ${g}_{u}$ obtained the highest ${\delta }_{1}$ indices. However, their importance drastically decreased in Step 2 (${\delta }_{2,{\mu }_{u}}$ = 0) when the correlations smoothed their effect on the model output. Conversely, ${k}_{2}$, with ${\delta }_{1,{k}_{2}}>0$ and the highest ${\delta }_{2},$ remained the most important parameter. Finally, the ‘indirect’ impact of the ${IC}_{50}$ was still present in the CV2 scenario as well as the moderate effect of ${m}_{u}$.

Results for the TVDT are illustrated in panels C and D of Fig. 5. ${\mu }_{u}$ and ${g}_{u}$ had the highest direct impact in both uncertainty scenarios. Again, although ${m}_{u}$ appears in the TVDT definition (Eq. (2)), its direct contribution was not relevant, in particular in the CV2 scenario where ${\delta }_{1,{m}_{u}}=0$. Again, ${\delta }_{Vmax}$, ${R}_{b}$, ${V}_{u10}$, ${W}_{0}$, ${IV}_{u50}$, ${k}_{2}$, ${IC}_{50}$ and ${k}_{1}$ had a ${\delta }_{1}$=0 because they did not appear in the equation of this metric. Among them, in both uncertainty case, ${\delta }_{Vmax}$ was the parameter with the highest ‘indirect’ effect due to its correlations.

### Results for ${\Delta T}_{{\delta }_{Vmax}}$

**Fig. 6:**
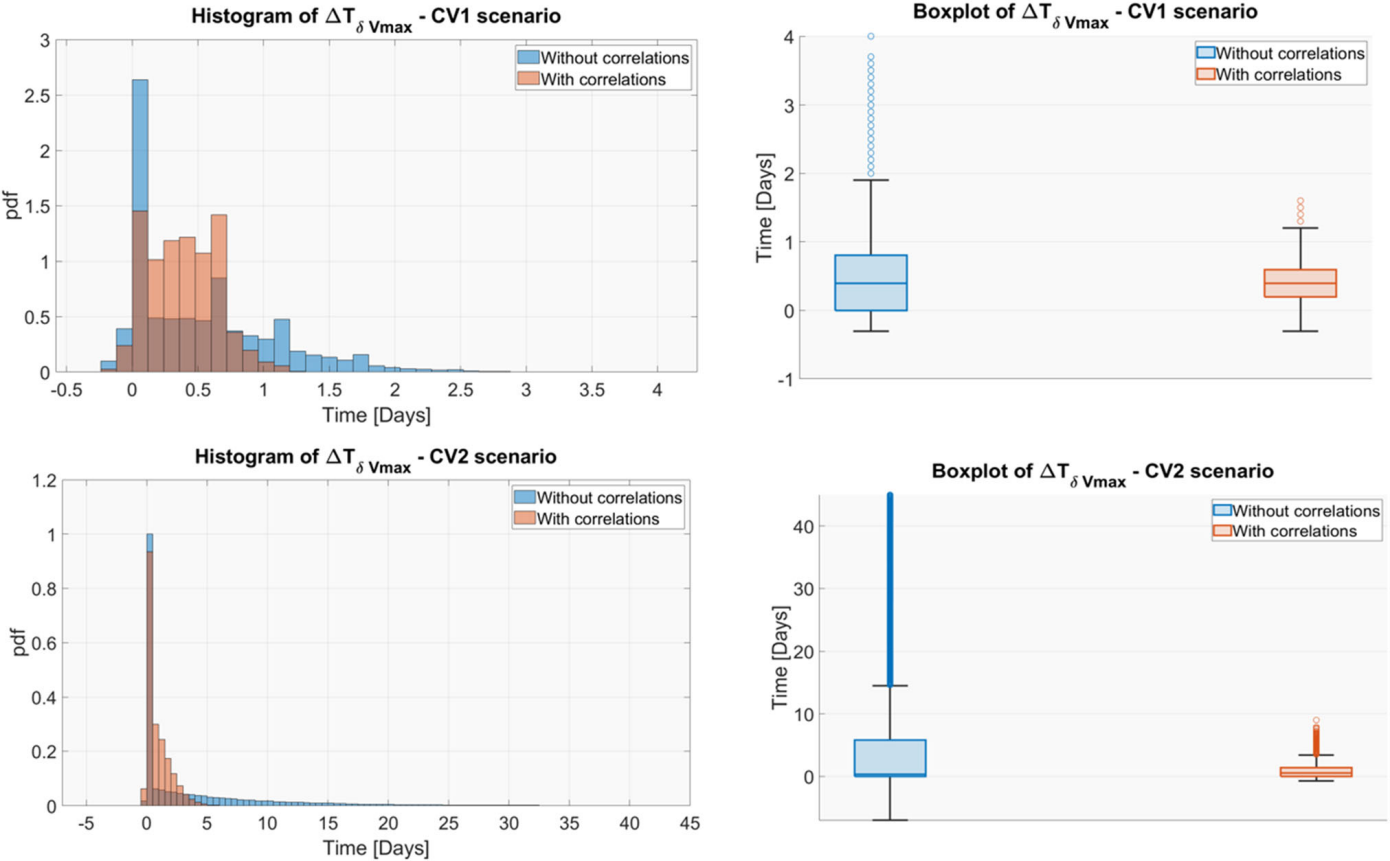
Histograms and box plots of ${\Delta \mathrm{T}}_{\mathrm{\delta Vmax}}$ considering and ignoring correlations for each uncertainty scenario

**Fig. 7:**
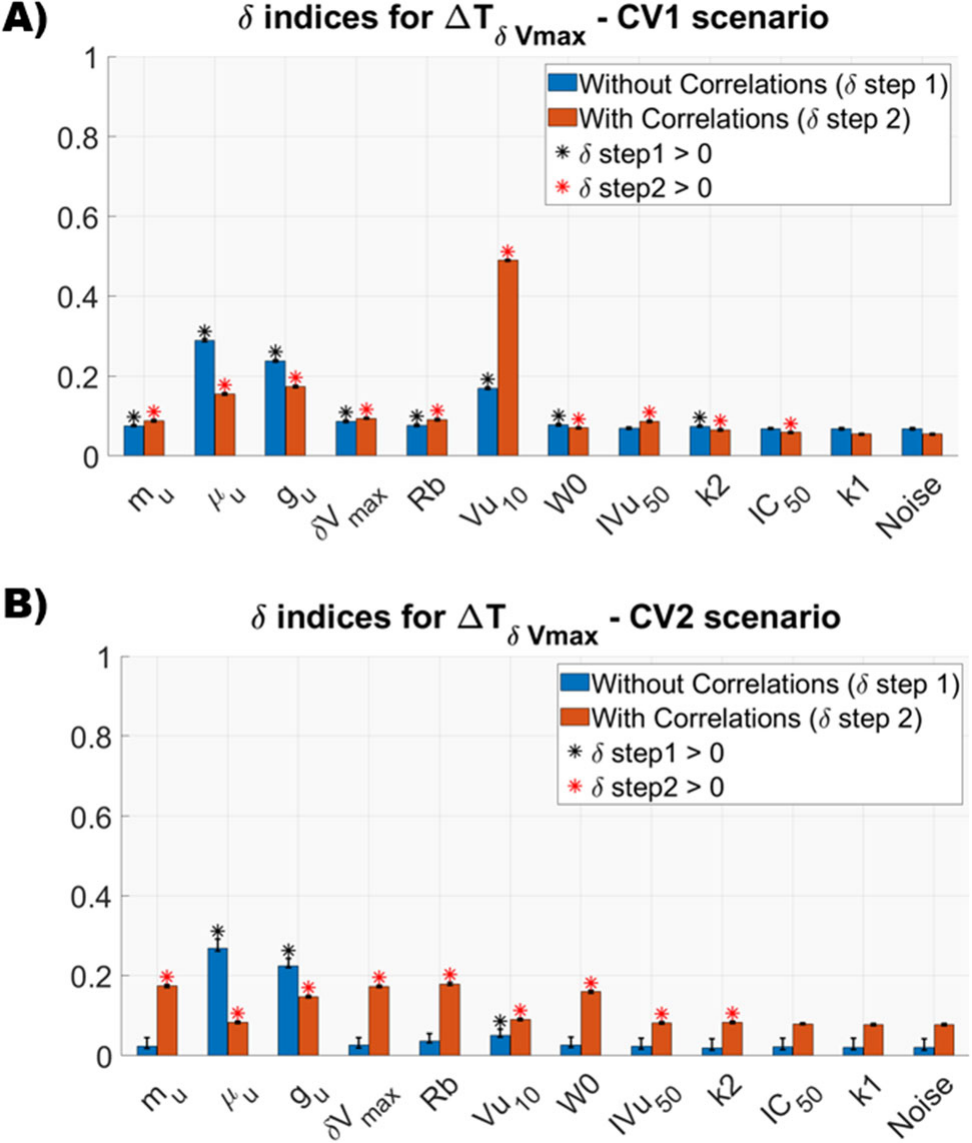
Results of the Two Stages GSA on the ${\Delta \mathrm{T}}_{\mathrm{\delta Vmax}}$ in both CV1 and CV2 scenario. Black and red asterisks mark parameters with a δ significatively greather than 0 in Step 1 and Step 2, respectively

The results of the uncertainty analysis are summarized in Fig. 6 where the distribution of ${\Delta T}_{{\delta }_{Vmax}}$ is represented through box plots and histograms, respectively. As already observed for ${C}_{T}$ and TVDT, also for ${\Delta T}_{{\delta }_{Vmax}}$ neglecting correlations between the parameters led to an overestimation of the output uncertainty, especially in the CV2 scenario. In the CV1 scenario the 95% CI of ${\Delta T}_{{\delta }_{Vmax}}$ was [− 0.10, 1.90] days when excluding and [− 0.10, 0.90] when including correlations, highlighting only a moderate overestimation of the variability in the first case. Conversely, in CV2 scenario (high uncertainty) the distribution of the ${\Delta T}_{{\delta }_{Vmax}}$ became more skewed and its 95% CI moved from [− 0.10, 3.10] to [0.00, 28.70] days when correlations were ignored. Differently to ${C}_{T}$ and TVDT, ${\Delta T}_{{\delta }_{Vmax}}$ range approximately remained within two folds of its mean only in CV1 scenario. This aspect made even more relevant performing a GSA to assess which uncertainty sources need to be reduced to obtain a more reliable estimation of the ${\Delta T}_{{\delta }_{Vmax}}.$ Thus, the two-stages GSA was applied to the ${\Delta T}_{{\delta }_{Vmax}}$ in both CV1 and CV2 case. The obtained results are reported in Fig. 7.

**Fig. 8:**
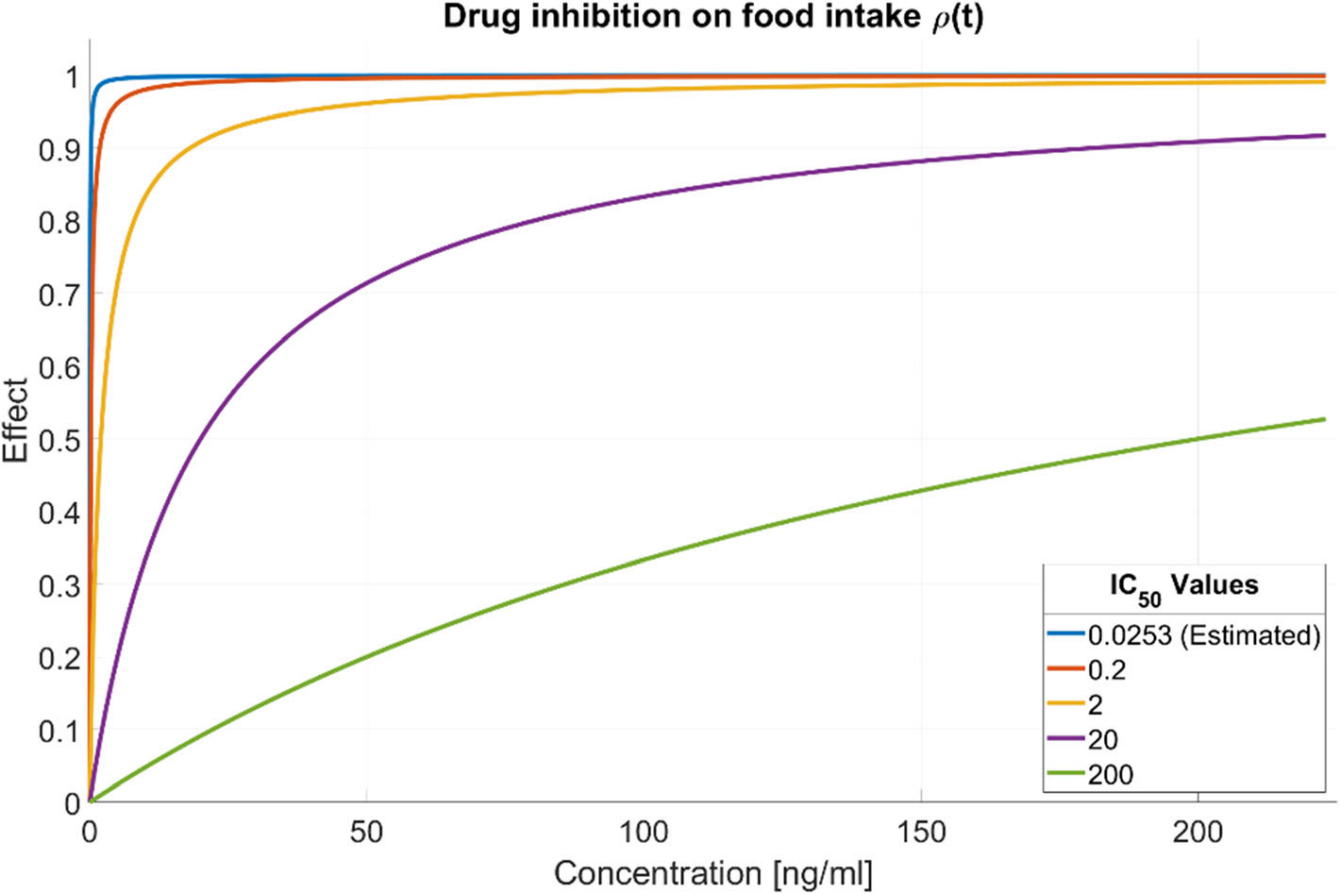
Levels of drug inhibition on food intake at different ${\mathrm{IC}}_{50}$ values. The blue line corresponds to the estimated value of this parameter. As the ${\mathrm{IC}}_{50}$ increases and reaches values in the order of magnitude of the drug plasma concentration, its effect on the inhibition of the food intake is more evident (Color figure online)

As the ${\Delta T}_{\delta Vmax}$ has no analytical definition, it is impossible to predict a priori which model parameters will not have or not a direct impact on the distribution of this metric. In the CV1 scenario (panel A of Fig. 7), ${\mu }_{u}$ and ${g}_{u}$ had the highest direct effect on the pdf of${\Delta T}_{\delta Vmax}$, followed by the initial tumor volume${V}_{u10}$. Differently, ${IV}_{u50},{IC}_{50}$ and ${k}_{1}$ had ${\delta }_{1}$ indices equal to 0 as their median δ were lower than the 97.5th percentile of negative control${\delta }_{1,Noise}$. Therefore, it was assessed that they had no causal impact on this output. Among them, only ${k}_{1}$ had a ${\delta }_{2}$=0, indicating that also its ‘indirect’ contribution was not relevant. The parameters with the highest ${\delta }_{2}$ indices resulted${V}_{u10}$,${g}_{u}$ and ${\mu }_{u}.$ As they also obtained a ${\delta }_{1}$>0, it was possible to conclude that they had both a direct and ‘indirect’ impact on the ${\Delta T}_{\delta Vmax}$ distribution $.$ In the scenario with higher CVs (panel B of Fig. 7), tumor growth related parameters, ${\mu }_{u}$ and ${g}_{u},$ had the highest causal effects. All the other parameters, except for the ${V}_{u10},$ had ${\delta }_{1}$ indices 0. From the Step 2, it emerged that, even if the effects of ${\mu }_{u}$ and ${g}_{u}$ were confirmed, the parameters with the highest impact due to correlations were${\delta }_{Vmax}$,${R}_{b}$,${W}_{0}$, ${m}_{u}.$ Differently, ${IC}_{50}$ and ${k}_{1}$ were not impactful even when considering the statistical dependencies. To understand the lack of effect exerted by ${IC}_{50}$ on the ${\Delta T}_{\delta Vmax}$ metric, it is important to observe that the estimated value of this parameter (Table 2) is three orders of magnitude smaller than the concentration level observed for the drug at the administered dose schedule (Table S1.1 of Supplementary Material S1). This implies that the inhibition of pharmacological treatment on food intake is always maximum for all the sampled values of IC_50_ as shown by Fig. 8.

## Discussions

Pharmacometric models play a key role within the process of anticancer drug development as they can help to inform future clinical trials (e.g., anticipation of the first in-human dose) starting from preclinical data [5, 6, 16, 18]. Therefore, it is essential to investigate the reliability of the model-based inference. Pharmacometric model parameters can be affected by different sources of uncertainty and/or variability. For example, high uncertainty can arise from the model identification on sparse experimental data. This uncertainty can be propagated to the model outputs and predictions. In this context, uncertainty analysis helps in understanding whether model predictions are too uncertain to be useful [30]. In addition, GSA is strongly recommended [30, 31] as it can provide useful insights on what are the parameters mostly responsible for the model output uncertainty.

The majority of GSA techniques relies on the assumption of independent model inputs [27, 36]. However, this hypothesis can be a strong limitation in many situations. For example, when the GSA scope is to investigate the impact of the estimation uncertainty, it is necessary to consider the correlation related to the estimation process. Ignoring known correlations between model inputs may dramatically alter the model simulations and, consequently, the GSA results [28, 31, 40, 44]. However, the field of GSA in presence of correlated inputs is still embryonic [31, 39–41].

In this paper, a novel framework for performing GSA in presence of correlations between model input parameters was proposed based on the $\delta$ sensitivity index. The choice of $\delta$ index was done as this index is well defined also in presence of statistical dependencies between model inputs and provides an easier interpretability of the results [45]. This new method was applied on the tumor-in-host DEB-TGI model [15, 20, 24] with the aim of evaluating the impact of estimate uncertainty on some relevant model-derived metrics. In particular, the focus was on the concentration threshold for tumor eradication, ${C}_{T},$ and the tumor volume doubling time, TVDT, (Eqs. (2) and (3)) derived by the stability analysis of the model [15]. In addition, the ${\Delta T}_{\delta Vmax}$, introduced in this paper, was considered. The analysis was performed in two different scenarios of uncertainty, CV1 (“low uncertainty”) and CV2 (“high uncertainty”).

First, a preliminary analysis, where the uncertainty of the model parameters had been propagated to the outputs of interest, was performed (Figs. 4 and 6 along with Figure S4. 1 in Supplementary Material S4). Results remarked the importance of considering the parameter correlations during the model simulations. Indeed, neglecting the statistical dependencies between model parameters led to an overestimation of the model outputs uncertainty, especially when the inputs were sampled from a very wide space (i.e., case of high parameter uncertainty, CV2 scenario). Differently, the presence of correlations imposed some constraints on the sampled parameters, preventing the extraction of implausible parameter sets and, consequently, reducing the presence of outliers in the distribution of the outputs. The uncertainty analysis allowed to assess that, in the realistic scenario of correlated parameters, the estimation uncertainty did not critically affect ${C}_{T}$ and TVDT that approximatively remained within the two folds of their respective means, even in the CV2 scenario. The ${\Delta T}_{\delta Vmax}$ metric resulted more sensitive to the parameter uncertainty, however its variation remained limited at least for moderate CVs of parameters (CV1 scenario).

After this preliminary step, the GSA workflow introduced in this paper was applied to the three outputs of interests. The proposed approach is composed of two steps. In the first one, δ indices are computed neglecting correlations between parameters to quantify the causal effects of the model parameters on the outputs. In the second step, a new set of δ indices is calculated to taking into account also the ‘indirect’ effects due to the correlations and the real distribution of the output. By comparing the δ values obtained in Step 1 and Step 2, it was possible to evaluate how much the presence of correlations can alter the impact of the parameters on the model outputs and, thus, change the results of the GSA.

A parameter with a low causal effect on the model output (Step 1) can gain relevance due to its correlations with the other model inputs (Step 2). The extreme example of this situation is represented by parameters with ${\delta }_{1}$ = 0 and ${\delta }_{2}$>0, i.e., parameters exerting only an indirect effect due to their correlation with other model inputs: this is the case of $I{C}_{50}$ with respect to the model output ${C}_{T}$ (Fig. 5, panels A and B). This was trivial as $I{C}_{50}$ does not appear in the ${C}_{T}$ definition (Eq. (3)). However, $I{C}_{50}$ is strongly correlated with the drug potency ${k}_{2}$ which, in turn, exerts a strong direct effect on ${C}_{T}$ in both the uncertainty scenarios. For this reason, in the Step 2 of the two-stage GSA, the $I{C}_{50}$ gained relevance emerging as the second most impactful parameter. Such observation has to be carefully considered in the strategies aiming to reduce the estimation uncertainty of${C}_{T}$. An accurate estimate of ${k}_{2}$, the most directly impacting parameter on ${C}_{T},$ may not be enough to decrease the output uncertainty, but it is also necessary to reduce the statistical dependencies of those parameters highly correlated with${k}_{2}$, such as $I{C}_{50}.$ For example in this specific case, an experimental design that includes a group of treated tumor-free animals could help to statistically disentangle the two drug-related parameters.

Conversely, the presence of statistical dependencies can mitigate the causal effect of some model parameters. This is the case of inputs with high ${\delta }_{1}$ index and significant lower ${\delta }_{2}$. An example is provided by the parameters ${\mu }_{u}$ and ${g}_{u}$. They had a high direct effect (i.e., high ${\delta }_{1,{\mu }_{u}}, {\delta }_{1,{g}_{u}}$) on ${C}_{T}$, TVDT and ${\Delta T}_{\delta Vmax}$ in the two uncertainty scenarios (Figs. 5 and 6), however, when correlations were considered (Step 2), their impact was remarkably reduced. This example further emphasizes that correlations must be considered in the GSA framework to avoid a wrong ranking of the parameters.

The proposed two-stages approach represents and appropriate methodology for ranking correlated input parameters according to their impact on the model outputs. Furthermore, it helps to discern and quantify the sources of their effects (i.e., direct or indirect). In particular, the obtained ranking identifies those parameters that mostly affect the output uncertainty and consequently require a better estimation (e.g., by using optimal design strategies [54]) to improve the precision of the model predictions. At the same time, parameters with an irrelevant influence on the outputs of interest can be recognized. Assessing the robustness of the model predictions with respect to highly uncertain parameters is extremely relevant when these parameters are affected by identification problems. For example, parameter ${m}_{u}$, representing the tumor maintenance cost, is generally estimated with low precision [15, 19] and, consequently, high CVs were considered in both the uncertainty scenarios. The GSA performed on the ${C}_{T}$ highlighted that, even if ${m}_{u}$ appears the formula of ${C}_{T}$, its contribution to the output uncertainty is always close to 0 (panels A and B of Fig. 5). Thus, it was assessed that the prediction of the ${C}_{T}$ is quite robust with respect to an inaccurate identification of ${m}_{u}$.

This work remarks the usefulness of the GSA which provides a diagnostic tool to evaluate the robustness of the inference process based on pharmacometric models [9]. Here, we proposed and applied a two-stages GSA based on the δ sensitivity indices to deal with the estimation uncertainty and its associated correlations. In particular, in this work, the statistical dependencies between model input parameters were fixed according to typical correlation matrices obtained during model identification. However, an additional layer of complexity could be added by considering the uncertainty on the correlations, which becomes random variables with a certain distribution. This would imply that the probability density function of model inputs is uncertain and, consequently, the GSA results (i.e., sensitivity indices computed for each input parameter) too. In such case, a nested two-stages GSA could be performed to quantify the impact of each correlation on every sensitivity index [55]. Unfortunately, this strategy is computationally demanding and difficult to implement with lots of uncertain parameters in the input distributions and in presence of complex model simulations [55].

Although the developed GSA method was applied to analyse the effects of estimation uncertainty and its associated correlations, it can be used to characterize different sources of variations. As an example, in the Supplementary Material S5 it was exploited to assess the impact of the inter-tumor cell line variability of the DEB-TGI parameters on the${C}_{T}$. Further, the investigation of the impact of the inter-drug variability on a single tumor cell line could be another interesting analysis to perform [56]. Note that, the same approach can be applied to PBPK models in which parameters are numerous, strongly correlated and generally affected by a significant uncertainty/variability. Therefore, the proposed GSA method is recommended in presence of strong correlations between the model parameters as long as a sampling strategy, both neglecting and considering statistical dependencies, can be implemented, for example by using the analytical definition of the joint pdf, by sampling from the Bayesian posterior distribution [57] or by using copulas [58]*.*

## Conclusions

GSA provides a valid diagnostic tool for supporting modelling activities especially in the oncology field where mathematical models are often used to make inference from preclinical to clinical setting. However, the development of GSA methods well-defined and easy-to-interpret also in presence of statistical dependencies between model parameters is still at its early stage. The aim of this work was to introduce a novel Two-Stages approach based on the δ sensitivity index to perform GSA in presence of correlated model inputs. The proposed workflow was used to characterize the impact of the estimation uncertainty on output metrics of a tumor-in-host DEB-TGI model. This approach helped to rank the parameters according to their impact on the output distribution, discerning whether a parameter mainly exerts a direct or ‘indirect’ effect. In this way, it was possible to identify uncertainties that should be necessarily reduced to obtain robust predictions for the outputs of interest.

## Supplementary Information

Below is the link to the electronic supplementary material.

Supplementary file1 (DOCX 599 kb)
