# Supplementary material for: A two-stages global sensitivity analysis by using the δ sensitivity index in presence of correlated inputs: application on a tumor growth inhibition model based on the dynamic energy budget theory
Source: J Pharmacokinet Pharmacodyn. 2023 Jul 9;50(5):395–409. doi: 10.1007/s10928-023-09872-w (PMC10460734; doi:10.1007/s10928-023-09872-w)
Supplement: Supplementary file 1 — Supplementary file1 (DOCX 599 kb) [file 10928_2023_9872_MOESM1_ESM.docx]

# Supplementary Material S1

Table S1.1 Setting of the simulations performed to compute the ${\Delta T}_{\delta Vmax}.$

| **Simulation Parameter** | **Value** |
| --- | --- |
| Final time | 800 days |
| Time step | 0.1 days |
| Amount of administrated drug | 0.5 mg/kg |
| Administration days | 8^th^ and 12^th^ |

***Example of NONMEM input dataset***

Table S1. 2 Example of NONMEM dataset for running the DEB-GSA model according to the provided script.

| C | ID | TIME | DV | MDV | EVID | CMT | AMT | parMU | parMUU | parGU | parDELTA | parVU10 | parW0 | parIVU50 | parK2 | parC50 | parK1 | parRHO |
| --- | --- | --- | --- | --- | --- | --- | --- | --- | --- | --- | --- | --- | --- | --- | --- | --- | --- | --- |
| . | 1 | 0 | . | 0 | 0 | 8 | . | 0.01 | 13.3 | 10.2 | 0.05 | 0.001 | 22.5 | 5.82 | 0.12 | 0.02 | 0.51 | 0.91 |
| . | 1 | 0.5 | . | 0 | 0 | 8 | . | 0.01 | 13.3 | 10.2 | 0.05 | 0.001 | 22.5 | 5.82 | 0.12 | 0.02 | 0.51 | 0.91 |
| . | 1 | 1 | . | 0 | 0 | 8 | . | 0.01 | 13.3 | 10.2 | 0.05 | 0.001 | 22.5 | 5.82 | 0.12 | 0.02 | 0.51 | 0.91 |
| . | 1 | 1.5 | . | 0 | 0 | 8 | . | 0.01 | 13.3 | 10.2 | 0.05 | 0.001 | 22.5 | 5.82 | 0.12 | 0.02 | 0.51 | 0.91 |
| . | 1 | 2 | . | 0 | 0 | 8 | . | 0.01 | 13.3 | 10.2 | 0.05 | 0.001 | 22.5 | 5.82 | 0.12 | 0.02 | 0.51 | 0.91 |
| . | 1 | 2.5 | . | 0 | 0 | 8 | . | 0.01 | 13.3 | 10.2 | 0.05 | 0.001 | 22.5 | 5.82 | 0.12 | 0.02 | 0.51 | 0.91 |
| . | 1 | 3 | . | 0 | 0 | 8 | . | 0.01 | 13.3 | 10.2 | 0.05 | 0.001 | 22.5 | 5.82 | 0.12 | 0.02 | 0.51 | 0.91 |
| . | 1 | 3.5 | . | 0 | 0 | 8 | . | 0.01 | 13.3 | 10.2 | 0.05 | 0.001 | 22.5 | 5.82 | 0.12 | 0.02 | 0.51 | 0.91 |
| . | 1 | 4 | . | 0 | 0 | 8 | . | 0.01 | 13.3 | 10.2 | 0.05 | 0.001 | 22.5 | 5.82 | 0.12 | 0.02 | 0.51 | 0.91 |
| . | 1 | 4.5 | . | 0 | 0 | 8 | . | 0.01 | 13.3 | 10.2 | 0.05 | 0.001 | 22.5 | 5.82 | 0.12 | 0.02 | 0.51 | 0.91 |
| . | 1 | 5 | . | 0 | 0 | 8 | . | 0.01 | 13.3 | 10.2 | 0.05 | 0.001 | 22.5 | 5.82 | 0.12 | 0.02 | 0.51 | 0.91 |
| . | 1 | 5.5 | . | 0 | 0 | 8 | . | 0.01 | 13.3 | 10.2 | 0.05 | 0.001 | 22.5 | 5.82 | 0.12 | 0.02 | 0.51 | 0.91 |
| . | 1 | 6 | . | 0 | 0 | 8 | . | 0.01 | 13.3 | 10.2 | 0.05 | 0.001 | 22.5 | 5.82 | 0.12 | 0.02 | 0.51 | 0.91 |
| . | 1 | 6.5 | . | 0 | 0 | 8 | . | 0.01 | 13.3 | 10.2 | 0.05 | 0.001 | 22.5 | 5.82 | 0.12 | 0.02 | 0.51 | 0.91 |
| . | 1 | 7 | . | 0 | 0 | 8 | . | 0.01 | 13.3 | 10.2 | 0.05 | 0.001 | 22.5 | 5.82 | 0.12 | 0.02 | 0.51 | 0.91 |
| . | 1 | 8 | . | 1 | 1 | 1 | 0.5 | 0.01 | 13.3 | 10.2 | 0.05 | 0.001 | 22.5 | 5.82 | 0.12 | 0.02 | 0.51 | 0.91 |
| . | 1 | 8.5 | . | 0 | 0 | 8 | . | 0.01 | 13.3 | 10.2 | 0.05 | 0.001 | 22.5 | 5.82 | 0.12 | 0.02 | 0.51 | 0.91 |

**NONMEM code for the DEB-TGI model**

Model parameters are passed in input as covariates and they are reported in the dataset.

$PROBLEM DEB-TGI model simulator

;;;;;;;;;;;;;;;;;;;;;;;;;;;;;;;;;;;;;;;;;;;;;;;;;;;;;;;;;;;;;;;;;;;;;;;;

$INPUT C ID TIME DV MDV EVID CMT AMT parMU parMUU parGU parDELTA

parVU10 parW0 parIVU50 parK2 parC50 parK1 parRHO

;;;;;;;;;;;;;;;;;;;;;;;;;;;;;;;;;;;;;;;;;;;;;;;;;;;;;;;;;;;;;;;;;;;;;;;;

$DATA dataset.csv IGNORE=C

;;;;;;;;;;;;;;;;;;;;;;;;;;;;;;;;;;;;;;;;;;;;;;;;;;;;;;;;;;;;;;;;;;;;;;;;

$SUBROUTINE ADVAN6 TOL=6

;;;;;;;;;;;;;;;;;;;;;;;;;;;;;;;;;;;;;;;;;;;;;;;;;;;;;;;;;;;;;;;;;;;;;;;;

$MODEL NCOMP=8

;pk 2 comp

COMP(1,DEFDOSE);observable/administration

COMP(2);peripheral

;deb-tgi

COMP(3) ; energy

COMP(4) ; structural biomass

COMP(5) ; proliferant

COMP(6) ; mortality chain (1)

COMP(7) ; mortality chain (2)

COMP(8) ; mortality chain (3)

;;;;;;;;;;;;;;;;;;;;;;;;;;;;;;;;;;;;;;;;;;;;;;;;;;;;;;;;;;;;;;;;;;;;;;;;

$PK

test = THETA(1)*ETA(1)

;pk parameters (fixed)

Vol = 2.235

k10 = 21.528

k12 = 100.32

k21 = 34.992

;deb paramters (fixed)

omg = 0.75;omega

ni = 1.2243

V1inf = 22.6

g_deb = 12.2

xi = 0.184

dv_deb = 1

dvu = 1

e0 = 1.3

;deb parameters (not fixed)

rhob = parRHO

mu_u = parMUU

gu = parGU

mu = parMU

deltamax = parDELTA

IVu50 = parIVU50

k1 = parK1

k2 = parK2

IC50 = parC50

W0 = parW0

Vu10 = parVu10

m = ni/((V1inf**(1/3))*g_deb)

strBio0 = W0/(dv_deb*(1+(e0*xi)))

;initial conditions - pk

A_0(1)=0 ;administration

A_0(2)=0 ;periph

;initial conditions - DEB

A_0(3)=e0 ;energy

A_0(4)=strBio0 ;structural biomass

A_0(5)=Vu10 ;proliferant

A_0(6)=0 ;mortality chain(1)

A_0(7)=0 ;mortality chain(2)

A_0(8)=0 ;mortality chain(3)

;;;;;;;;;;;;;;;;;;;;;;;;;;;;;;;;;;;;;;;;;;;;;;;;;;;;;;;;;;;;;;;;;;;;;;;;

$DES

;pk ODEs

derV = 0

DADT(1) = -(k10+k12)*A(1) + k21*A(2)

DADT(2) = k12*A(1) - k21*A(2)

;check for avoiding numerical inconsistence with concentration values

IF(A(1).GT.0) THEN

CP = A(1)*1000/Vol; plasma concentration

ELSE

CP = 0

ENDIF

;nonmem cannot handle complex number. put some flag for identifying that situations in which

;a complex number

IF(A(4)<0.OR.(A(4)+A(5))<0)THEN

flagNeg = 1

ELSE

flagNeg = 0

ENDIF

;time dependent quantities

rho = rhob*(1-(A(5)/(IVu50+A(5))))*(1-(CP/(IC50+CP)))

ku = (mu_u*A(5))/(A(4)+(mu_u*A(5)))

IF(flagNeg==0) THEN

flag1 = ((1-ku)*ni*A(3)*(A(4)**(2/3)) - (g_deb*m*A(4)))/(g_deb+((1-ku)*A(3)))

DADT(3) = (ni/(A(4)**(1/3)))*((rho*((V1inf/(A(5)+A(4)))**(2/3)))-A(3))

ELSE

flag1 = -999

DADT(3)= 0

ENDIF

IF(flagNeg==0.AND.flag1>=0) THEN

DADT(4) = (((1-ku)*ni*A(3)*(A(4)**(2/3))) - (g_deb*m*A(4)))/(g_deb+((1-ku)*A(3)))

derV = (((1-ku)*ni*A(3)*(A(4)**(2/3))) - (g_deb*m*A(4)))/(g_deb+((1-ku)*A(3)))

DADT(5) = ((((ni*A(4)**(2/3))+m*A(4))*g_deb*ku*A(3))/(g_deb*gu+((1-ku)*gu*A(3)))) - mu*A(5) - k2*A(5)*CP

ENDIF

IF(flagNeg==0.AND.flag1<0) THEN

flag2 = (((1-ku)*ni*A(3)*A(4)**(2/3)) - (g_deb*m*A(4)))/((1-ku)*(A(3)+(omg*g_deb)))

ELSE

flag2 = 999

ENDIF

IF(flag1<0.AND.flagNeg==0.AND.flag2<0.AND.flag2>=(-deltamax)) THEN

DADT(4) = (((1-ku)*ni*A(3)*A(4)**(2/3)) - (g_deb*m*A(4)))/((1-ku)*(A(3)+(omg*g_deb)))

derV = (((1-ku)*ni*A(3)*A(4)**(2/3)) - (g_deb*m*A(4)))/((1-ku)*(A(3)+(omg*g_deb)))

DADT(5) = (((m*g_deb*mu_u/gu) - mu) * A(5)) - k2*CP*A(5)

ENDIF

flagVmax = 0; flag in order to determinate the starting moment of Biomass degradation at maximum rate

IF(flag1<0.AND.flagNeg==0.AND.flag2<0.AND.flag2.LT.(-deltamax)) THEN

flagVmax= 1; activate flag

DADT(4) = -deltamax

derV = -deltamax

DADT(5) = (ku/gu)*( (A(3)*ni*A(4)**(2/3)) + deltamax*(A(3)+(omg*g_deb)) ) - mu*A(5) - k2*CP*A(5)

ENDIF

IF(flagNeg==1) THEN

DADT(1) = 0

DADT(2) = 0

DADT(3) = 0

DADT(4) = 0

DADT(5) = 0

DADT(6) = 0

DADT(7) = 0

DADT(8) = 0

ELSE

DADT(6) = k2*CP*A(5) - k1*A(6)

DADT(7) = k1*A(6) - k1*A(7)

DADT(8) = k1*A(7) - k1*A(8)

ENDIF

$ERROR

;output definition

q1 = A(1)

q2 = A(2)

energy = A(3)

strBio = A(4)

Vu1 = A(5)

Vu2 = A(6)

Vu3 = A(7)

Vu4 = A(8)

Worg = dv_deb*(1+(xi*energy))*strBio; body weight

Wtum = dvu*(Vu1+Vu2+Vu3+Vu4); tumor weight

Y = EPS(1)

$THETA ;typical values

; test

1

$OMEGA ;inter-individual variability

0.2 ;test

$SIGMA

1

$SIMULATION (38495) NSUB=1 ONLYSIMULATION

$TABLE ID TIME Worg Wtum energy strBio rho CP MDV flagVmax flag2 derV NOAPPEND NOPRINT ONEHEADER FILE=sdtab1

# Supplementary Material S2

Let us consider the following linear model

$$Y=100*X_{1}+100*X_{2}+{10*X}_{3}$$

(S2. 1)

with $X_{i}\sim N(0,1)$ and let $X_{4}$ be a spurious term (i.e., not directly appearing in the output equation) correlated to $X_{1}$ with $\rho_{1,4}=0.9.$ Figure S2. 1 and Figure S2. 2 illustrate the results of the GSA with the δ index computed neglecting and including correlations, respectively.


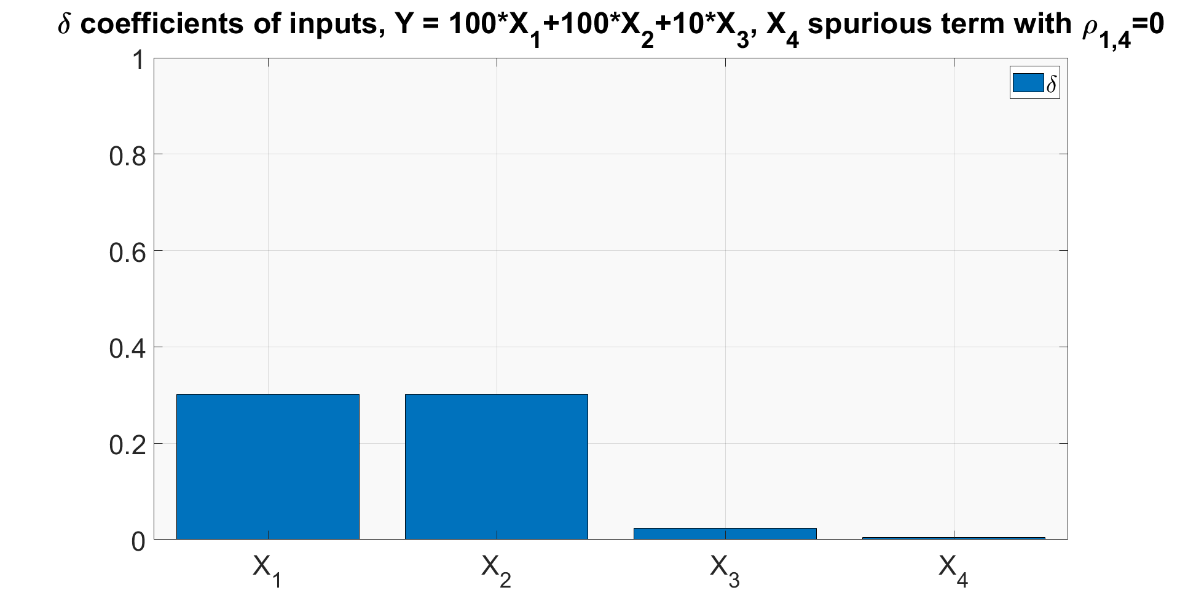


Figure S2. 1 Results of the GSA with the δ index on the model in Eq. (S2.1). Correlations between model inputs and the spurious term were not considered in the analysis in order to detect only the inputs with a causal impact on the output distribution.


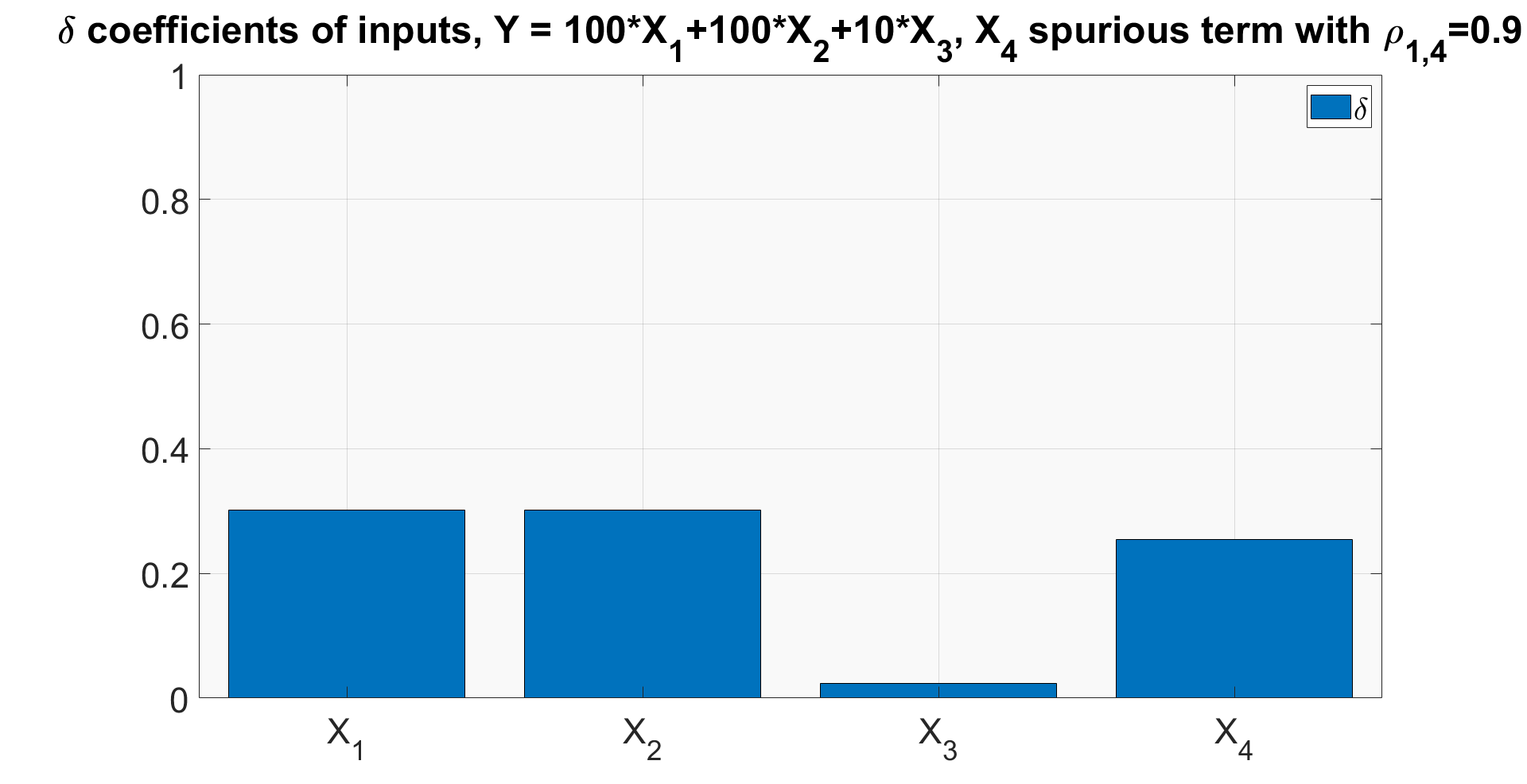


Figure S2. 2 Results of the GSA with the δ index on the model in Eq. (S2.1). Correlations between model inputs and the spurious term were considered in the analysis.

# Supplementary Material S3

Table S3. 1 DEB-TGI and PK model parameters assumed to be known without uncertainty/variability.

| **Parameter** | **Value** | **Unit** |
| --- | --- | --- |
| **Host-related parameters** | | |
| $\nu$ | 1.224 | $cm/day$ |
| $V_{1\infty}$ | 22.600 | $cm^{3}$ |
| $g$ | 12.200 | - |
| $\xi$ | 0.184 | - |
| $d_{V}$ | 1.000 | - |
| **Tumor-related parameters** | | |
| $d_{Vu}$ | 1.000 | - |
| **Cachexia-related parameters** | | |
| $\omega$ | 0.750 | - |
| **PK model parameters** | | |
| $V$ | 2.235 | $L/Kg$ |
| $k_{el}$ | 21.528 | $1/day$ |
| $k_{12}$ | 100.320 | $1/day$ |
| $k_{21}$ | 34.992 | $1/day$ |
| **Initial Conditions** | | |
| $e_{0}$ | 1.300 | - |

## Correlation structures of the DEB-TGI model parameters used in the Two-Stages GSA


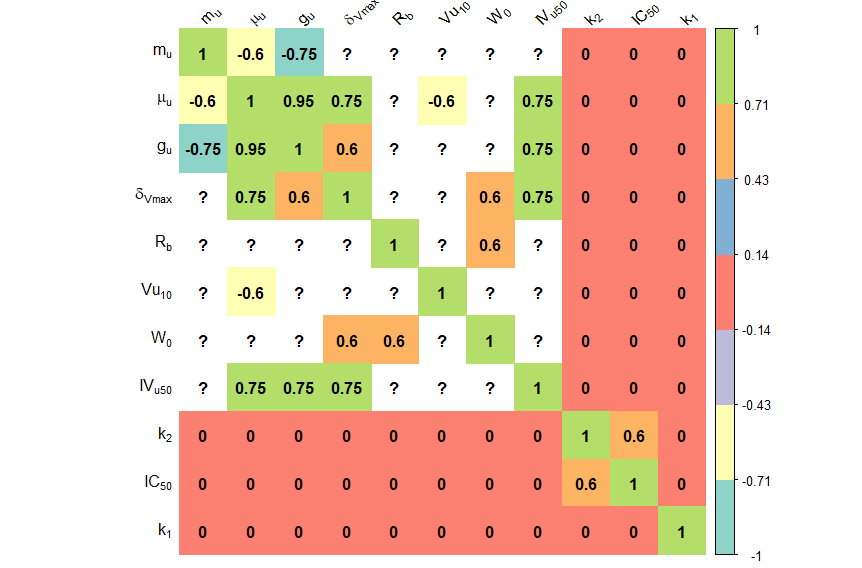


Figure S3. 1 Correlation structure between DEB-TGI parameters defined according to covariance matrix of estimates obtained from different model fitting on several preclinical datasets. Question marks represent the unknown correlations.

Correlations between model parameters were introduced based on typical correlation matrices of the parameter estimates obtained during the model identification step of several experiments (internal data, not shown). Based on these data, parameter couples were classified in significantly correlated (three level, i.e. corr = +/- 0.95,0.75 and 0.6), clearly uncorrelated (corr= 0) or with an unknown correlation as illustrated in Figure S3. 1. In particular, the last class included parameters couples for which correlations spanned over a wide range of values in the considered set of experiments.

The unknown correlations of the matrix reported in Figure S3. 1 were subsequently estimated by using the function *estimate_indirect_corr* of the mvLognCorrEst R package [1]. The complete correlation matrix is reported inFigure 3. The obtained correlation structure was combined with the typical values, **θ**, and the CVs of the model parameters (Table 2) to compute the covariance matrix **Ω** in both scenario (i.e., low (CV1) and high (CV2) variability). Then, the lognormally distributed parameter sets of the model were sampled from $LogN\left( \boldsymbol{\theta},\boldsymbol{\Omega} \right)$distribution.

More in details, the sampling strategy was based on reconducting the extraction to the underlying multivariate Normal distribution $N\left( \boldsymbol{\mu},\boldsymbol{\Sigma} \right)$, where **μ** is the vector of the means and **Σ** the normal covariance matrix, of $LogN\left( \boldsymbol{\theta},\boldsymbol{\Omega} \right)$. Once a parameter set $\boldsymbol{p}_{\boldsymbol{i}}$ is extracted from $N\left( \boldsymbol{\mu,\Sigma} \right)$, then it is possible to come back to the lognormal domain with an exponential transformation. $\boldsymbol{\mu}$ and $\boldsymbol{\Sigma}$ are computed from **θ** and **Ω** of the lognormal distribution by applying Eqs. (S3. 1) and (S3. 2)

$$\Sigma_{ij}=\ln\left( \frac{\Omega_{ij}}{\theta_{i}*\theta_{j}}+1 \right)$$

(S3. 1)

$$\mu_{i}=\ln\theta_{i}+\frac{1}{2} \Sigma_{ii}$$

(S3. 2)

In the CV2 scenario, the presence of higher CVs and the imposed correlation structure (Figure 3) violated some theoretical constraints of the LogNormal distribution [2-4]. In particular, by applying Eq.(S3. 1) on the lognormal covariance matrix **Ω**, it was obtained a normal covariance matrix **Σ** which was not positive semidefinite.

In this case, **Σ** was approximated with its nearest positive definite matrix, $\hat{\boldsymbol{\Sigma}}$, using Frobenius norm as matrix distance. $\hat{\boldsymbol{\Sigma}}$was computed from **Σ** with the method illustrated in [5,6]. The introduction of this approximation allowed to sample lognormally distributed parameter sets also for the CV2 scenario. However, the substitution of **Σ** with $\hat{\boldsymbol{\Sigma}}$ altered the original lognormal correlation structure of the model parameters (Figure 3). Thus, the correlation matrix reported in Figure S3. 2 was used in the CV2 scenario. It is important to underline that the effect of the approximation was very low, in fact the ratio between $\left\| \boldsymbol{\Sigma}\mathbf{-}\hat{\boldsymbol{\Sigma}} \right\|$ and $\left\| \boldsymbol{\Sigma} \right\|$ was 0.045, where $\left\| \cdot\right\|$ stands for the Frobenius Norm.


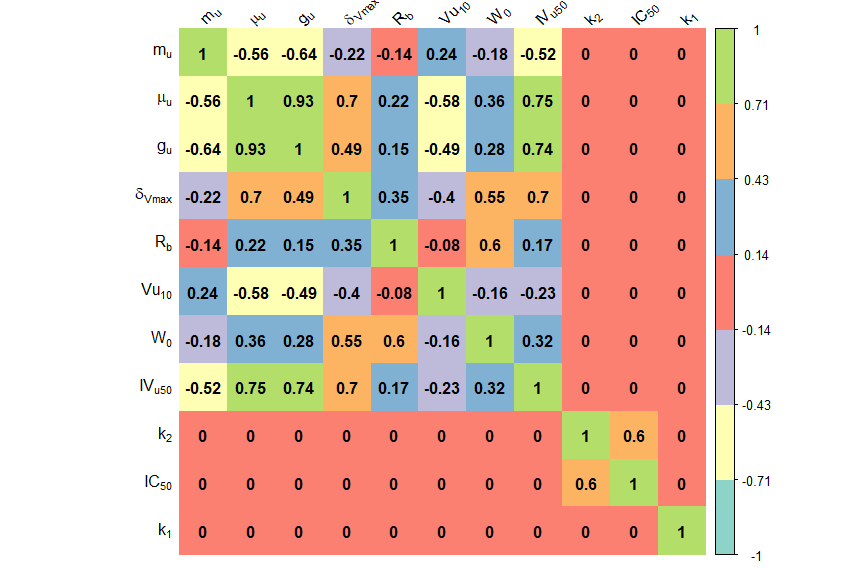


Figure S3. 2 : Correlation structure used in the CV2 scenario. The approximation introduced due to the violation of theoretical bounds of the multivariate Log-Normal distribution brought some differences in comparison with the original correlation structure illustrated in Figure 3.

1. De Carlo A, Tosca EM, Melillo N *et al.* mvLognCorrEst: an R package for sampling from multivariate lognormal distributions and estimating correlations from uncomplete correlation matrix. *Computer Methods and Programs in Biomedicine* 2023;**235**:107517.

2. Žerovnik G, Trkov A, Kodeli IA *et al.* Random Sampling of Correlated Parameters – a Consistent Solution for Unfavourable Conditions. *Nuclear Data Sheets* 2015;**123**:185–90.

3. Xavier HS, Abdalla FB, Joachimi B. Improving lognormal models for cosmological fields. *Mon Not R Astron Soc* 2016;**459**:3693–710.

4. Žerovnik G, Trkov A, Smith DL *et al.* Transformation of correlation coefficients between normal and lognormal distribution and implications for nuclear applications. *Nuclear Instruments and Methods in Physics Research Section A: Accelerators, Spectrometers, Detectors and Associated Equipment* 2013;**727**:33–9.

5. Higham NJ. Computing the nearest correlation matrix--a problem from finance. *IMA Journal of Numerical Analysis* 2002;**22**:329–43.

6. Higham NJ. Computing a nearest symmetric positive semidefinite matrix. *Linear Algebra and its Applications* 1988;**103**:103–18.

# Supplementary Material S4


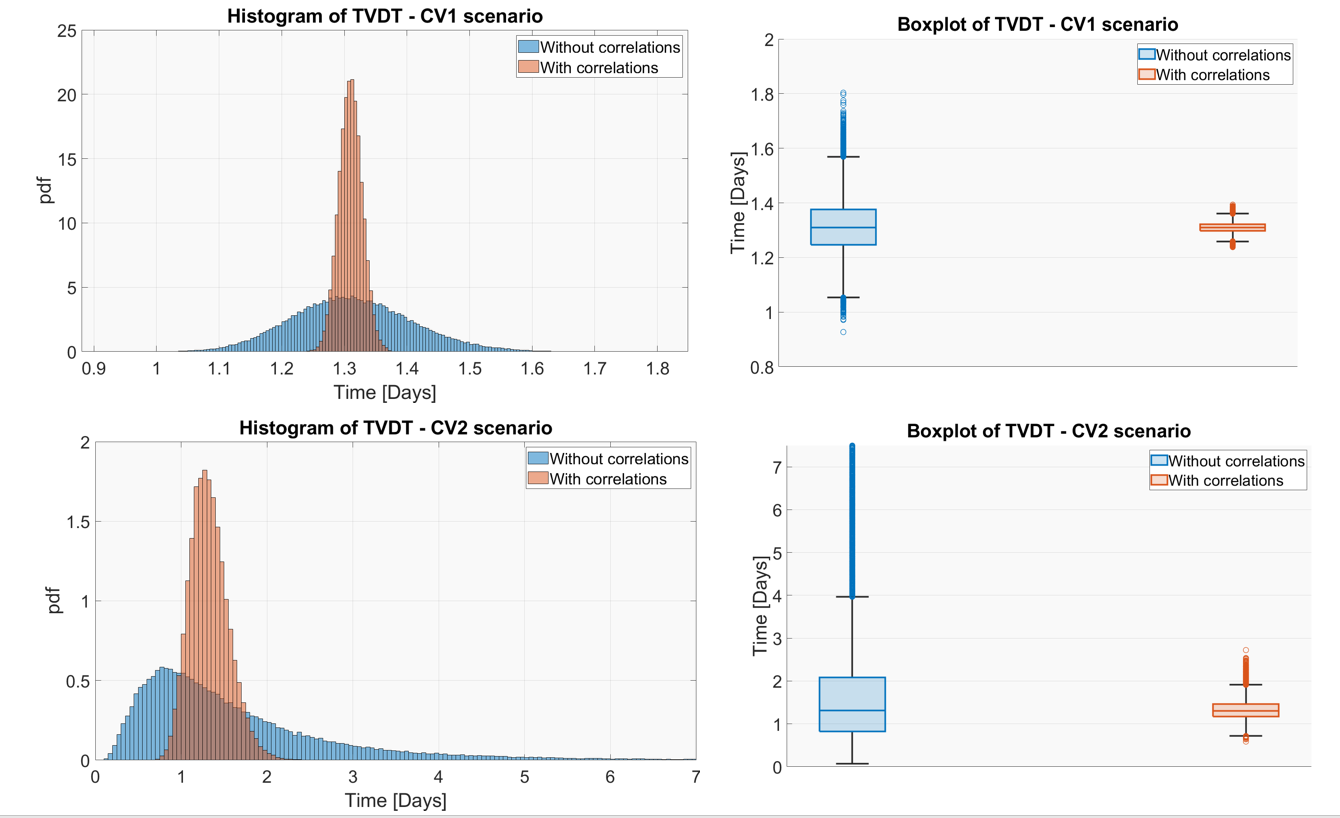


Figure S4. 1 Histograms and box plots of TVDT considering and ignoring correlations for each scenario.

# Supplementary Material S5

The two-stages GSA proposed in this work was applied on the tumor-in-host DEB-TGI model to evaluate the impact of the variability of the model parameters between different tumor cell lines on the threshold concentration for tumor eradication, $C_{T}$.

## S5.1 Definition of the parameter distribution

The parameters considered in this analysis are the same already used and reported in the main (Table 2). The preliminary step is the definition of the joint pdf. Different set of parameters obtained form xenograft experiments conducted on different tumor cell lines were considered. A uniform or log-uniform distribution was assumed for each model parameter The maximum and minimum values obtained in the considered experiments provided the range of the distribution. Table S5. 1 summarizes the assumptions.

Table S5. 1 Ranges and distributions of the the DEB-TGI parameters used in the two-stages GSA to assess the impact of inter- tumor cell line variability on $C_{T}.$

| **Parameter** | **Range** | **Unit** | **Distribution** |
| --- | --- | --- | --- |
| $\mu_{u}$ | 1.770 – 5.600 | - | Uniform |
| $g_{u}$ | 8.280 – 11.200 | - | Uniform |
| $m_{u}$ | 0.021 – 0.041 | $1/day$ | Uniform |
| $\delta_{Vmax}$ | 0.010 – 0.067 | - | Uniform |
| $V_{u10}$ | 0.005 – 0.047 | $g$ | Uniform |
| $W_{0}$ | 16.500 – 25.300 | $g$ | Uniform |
| $R_{b}$ | 0.000 – 0.236 | - | Uniform |
| $IV_{u50}$ | 1.510 – 66.100 | $cm^{3}$ | Uniform |
| $k_{1}$ | 0.038 – 0.936 | $1/day$ | Uniform |
| $k_{2}$ | 4.940e^-4^  - 0.0142 | $ml/ng day$ | Loguniform |
| $IC_{50}$ | 4.730e^-16^  - 4.150e^-4^ | $ng/ml$ | Loguniform |

Figure S5.1 shows the correlation matrix between model parameters, identified form the various experimental arms.


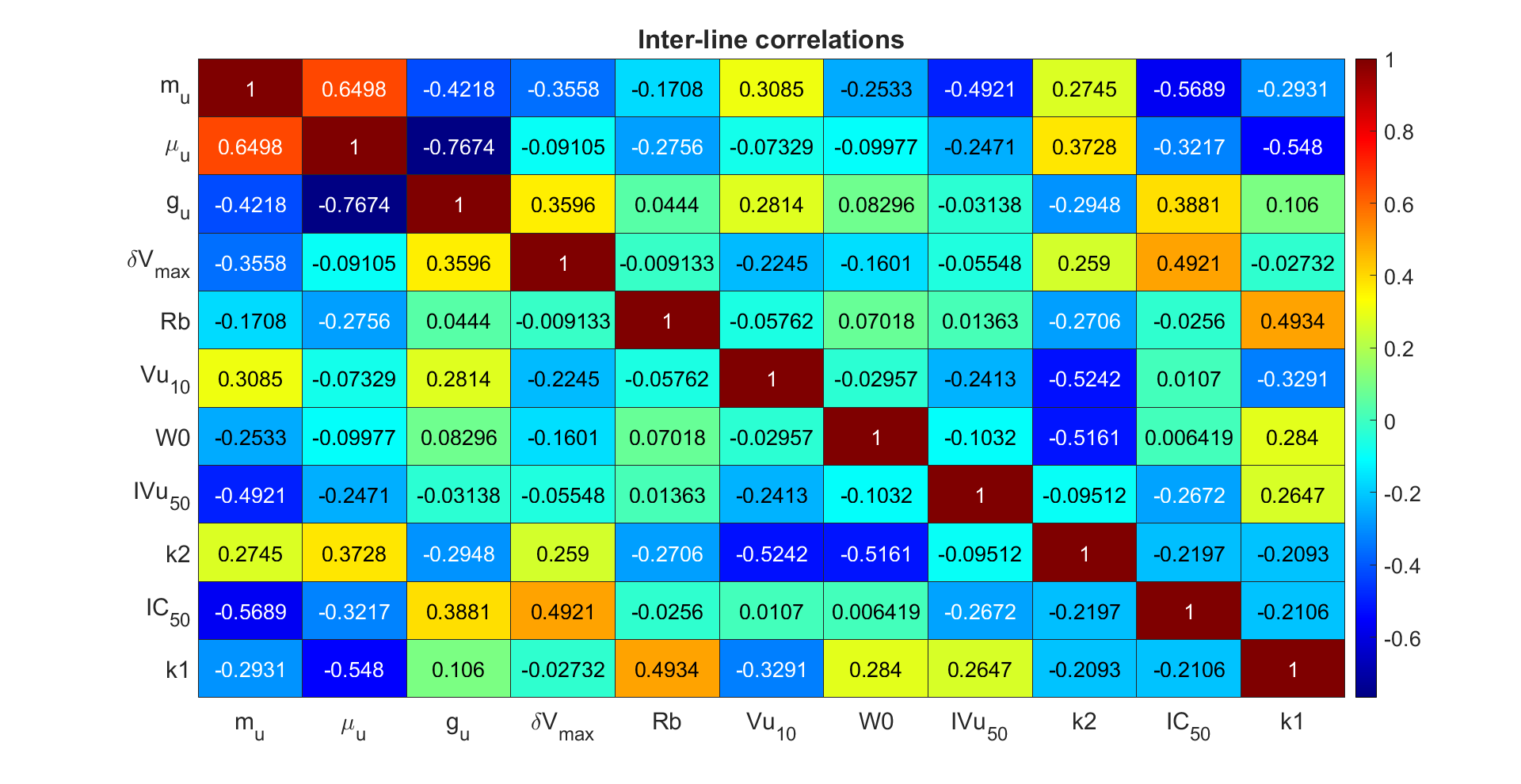


Figure S5.1 Correlation matrix used in the two-stages GSA to assess the impact of the inter-tumor cell line variability to the distribution of $C_{T}$.

## S5.2 Results of the Two-Stages GSA on the $C_{T}$

Figure S5.2 illustrates the results obtained by the two-stages GSA. All parameters appearing in the $C_{T}$ equation (Eq.3) had a direct impact that significantly differs from 0. In particular, the $k_{2}$ topped the Step 1 with the highest δ; same results in the Step 2. Among the parameters that had δ_1_=0, ${Vu}_{10}$ and $W_{0}$ gained a moderate effect on the distribution of this metric due to their correlations. Thus, from this analysis it was possible to conclude that drug potency was the most impactful parameter on the $C_{T}$ when the inter-line variability was considered.


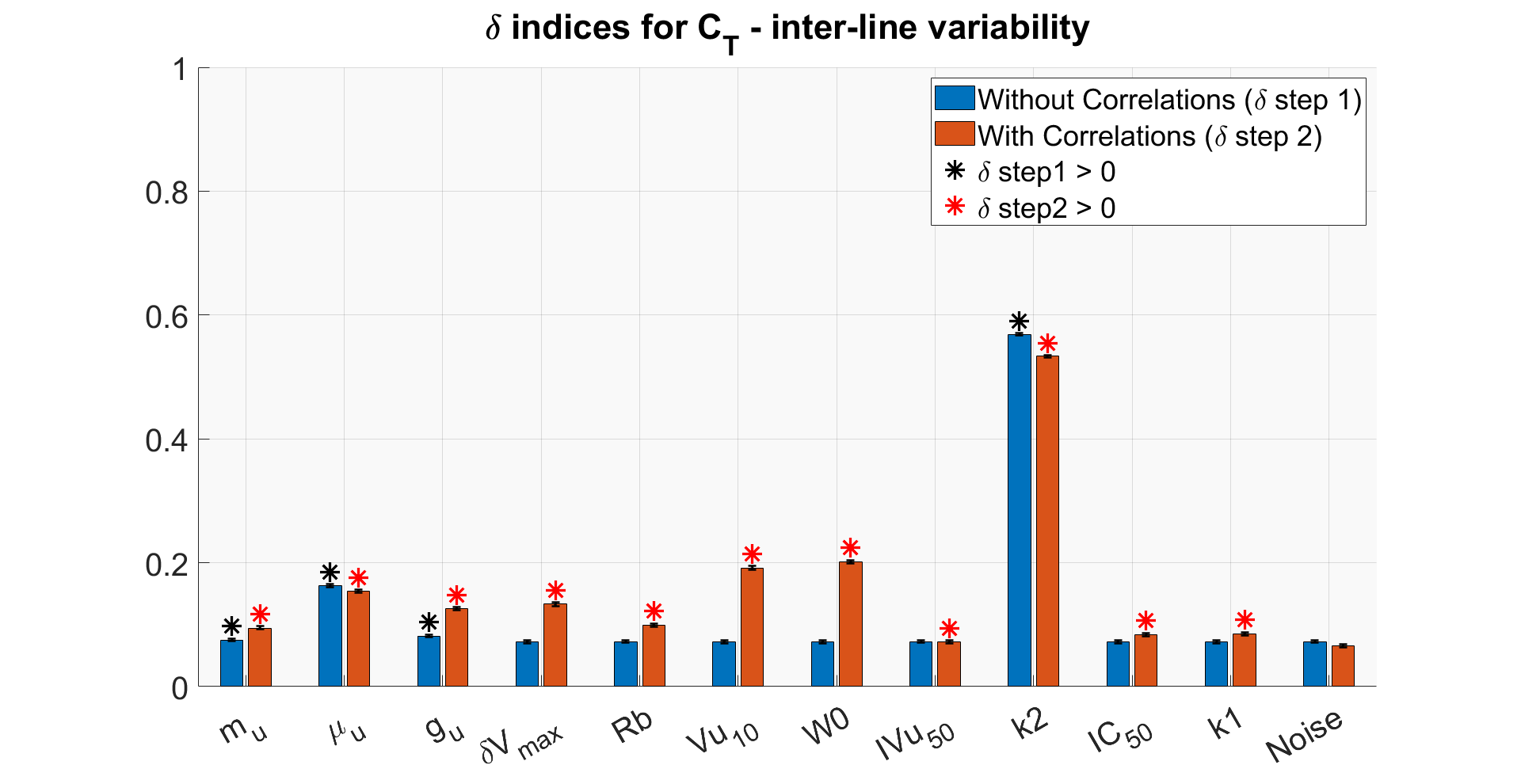


Figure S5.2 Results of the Two-Stages GSA on $C_{T}$ considering the inter-line variability. Black asterisks mark parameters with a δ significatively grather than 0 in Step 1 and Step2, respectively.
